# AltitudeOmics: The Integrative Physiology of Human Acclimatization to Hypobaric Hypoxia and Its Retention upon Reascent

**DOI:** 10.1371/journal.pone.0092191

**Published:** 2014-03-21

**Authors:** Andrew W. Subudhi, Nicolas Bourdillon, Jenna Bucher, Christopher Davis, Jonathan E. Elliott, Morgan Eutermoster, Oghenero Evero, Jui-Lin Fan, Sonja Jameson-Van Houten, Colleen G. Julian, Jonathan Kark, Sherri Kark, Bengt Kayser, Julia P. Kern, See Eun Kim, Corinna Lathan, Steven S. Laurie, Andrew T. Lovering, Ryan Paterson, David M. Polaner, Benjamin J. Ryan, James L. Spira, Jack W. Tsao, Nadine B. Wachsmuth, Robert C. Roach

**Affiliations:** 1 Altitude Research Center, Department of Emergency Medicine, University of Colorado Anschutz Medical Campus, Aurora, Colorado, United States of America; 2 Department of Biology, University of Colorado Colorado Springs, Colorado Springs, Colorado, United States of America; 3 Institute of Sports Sciences and Department of Physiology, Faculty of Biology and Medicine, University of Lausanne, Lausanne, Switzerland; 4 Department of Human Physiology, University of Oregon, Eugene, Oregon, United States of America; 5 Lemanic Doctoral School of Neuroscience, University of Lausanne, Lausanne, Switzerland; 6 AnthroTronix, Inc., Silver Spring, Maryland, United States of America; 7 Departments of Anesthesiology and Pediatrics, University of Colorado School of Medicine and Children's Hospital Colorado, Aurora, Colorado, United States of America; 8 Department of Integrative Physiology, University of Colorado Boulder, Boulder, Colorado, United States of America; 9 United States Department of Veterans Affairs, National Center for PTSD, Pacific Islands Health Care System, and Department of Psychiatry, University of Hawaii John A. Burns School of Medicine, Honolulu, Hawaii, United States of America; 10 Wounded, Ill & Injured Directorate (M9), United States Navy Bureau of Medicine and Surgery, Falls Church, Virginia, United States of America; 11 Department of Sports Medicine/Sports Physiology, University of Bayreuth, Bayreuth, Germany; Oregon Health & Science University, United States of America

## Abstract

An understanding of human responses to hypoxia is important for the health of millions of people worldwide who visit, live, or work in the hypoxic environment encountered at high altitudes. In spite of dozens of studies over the last 100 years, the basic mechanisms controlling acclimatization to hypoxia remain largely unknown. The AltitudeOmics project aimed to bridge this gap. Our goals were 1) to describe a phenotype for successful acclimatization and assess its retention and 2) use these findings as a foundation for companion mechanistic studies. Our approach was to characterize acclimatization by measuring changes in arterial oxygenation and hemoglobin concentration [Hb], acute mountain sickness (AMS), cognitive function, and exercise performance in 21 subjects as they acclimatized to 5260 m over 16 days. We then focused on the retention of acclimatization by having subjects reascend to 5260 m after either 7 (n = 14) or 21 (n = 7) days at 1525 m. At 16 days at 5260 m we observed: 1) increases in arterial oxygenation and [Hb] (compared to acute hypoxia: PaO_2_ rose 9±4 mmHg to 45±4 while PaCO_2_ dropped a further 6±3 mmHg to 21±3, and [Hb] rose 1.8±0.7 g/dL to 16±2 g/dL; 2) no AMS; 3) improved cognitive function; and 4) improved exercise performance by 8±8% (all changes p<0.01). Upon reascent, we observed retention of arterial oxygenation but not [Hb], protection from AMS, retention of exercise performance, less retention of cognitive function; and noted that some of these effects lasted for 21 days. Taken together, these findings reveal new information about retention of acclimatization, and can be used as a physiological foundation to explore the molecular mechanisms of acclimatization and its retention.

## Introduction

Millions of people live and work in, or travel to, high altitudes, and many of them are able to adjust successfully to the hypoxic environment of very high altitudes (∼5000 m), where ambient oxygen pressure is about half the sea level value. Discovery of the mechanisms responsible for human acclimatization to hypoxia could lead to new ways to improve acclimatization and its retention.

The physiology of how humans respond acutely and adapt to hypoxia has been explored extensively over the last century, yet many questions remain about the attributes that best characterize acclimatization [1]. Most would agree that improving arterial oxygenation and exercise performance are central tenets of acclimatization, and although no studies have focused on the protection from high-altitude illness that occurs with acclimatization, most would also agree such protection is an important aspect of acclimatization. On the other hand, how cognitive function responds during acclimatization is largely unknown, except from anecdotal reports. Intriguing also are suggestions that acclimatization causes functional modifications that persist upon return to high altitude after weeks, or perhaps even months, at sea level, and at a time when all known physiological measures of acclimatization have returned to normal low altitude values [2]–[4].

AltitudeOmics is a multifaceted research program on acclimatization to high altitude and the retention of acclimatization after return to low altitude. The goals for AltitudeOmics were 1) to describe a phenotype for successful acclimatization and assess its retention—that is—whether adaptive responses persist after descent to low altitude for one to three weeks, and 2) to use these findings as a foundation for companion mechanistic studies of the human transcriptome, epigenome, metabolome, and proteome (OMICS). Our approach was to study lowland volunteers in the field who were taken rapidly to 5260 m, where they acclimatized for 16 days. They then descended to 1525 m for either seven (n = 14) or 21 (n = 7) days, after which they returned quickly to 5260 m and were retested. This report describes the physiology of acclimatization and its retention for four key features of acclimatization: 1) arterial oxygenation and [Hb]; 2) acute mountain sickness (AMS); 3) cognitive function; and 4) exercise performance. Of particular interest was the acclimatization retention displayed upon returning to 5260 m after even three weeks at low altitude. Subsequent reports will explore changes in OMICS responses and will attempt to link those responses to the physiological phenotype of acclimatization and its retention reported here.

## Methods

### Ethical Approval and Subject Recruitment

The study was performed according to the Declaration of Helsinki. It was approved by the Institutional Review Boards of the Universities of Colorado and Oregon and by the Human Research Protection Office of the U.S. Department of Defense. The subjects were informed about the possible risks and discomforts of participation in the study before giving their written and verbal consent to participate. Physical examinations and the U.S. Army Physical Fitness Test (APFT) (push-ups, sit-ups, and a 3.2-km run) [5] were performed to characterize health and fitness status. Exclusion criteria included: being born at >1500 m; having traveled to altitudes >1000 m in the past three months (including air travel); using prescription medications; smoking; being pregnant or lactating; having a history of serious head injury (loss of consciousness); self or familial history of migraine; known hematologic or cardiovascular abnormality (e.g., sickle cell trait, cardiac arrhythmia); pulmonary function or diffusion capacity for carbon monoxide <90% of predicted; or failure to meet the minimal age/gender standards for the APFT [5]. Seventy-nine subjects completed the screening. Twenty-four healthy, physically active subjects were enrolled. Two subjects dropped out for non-altitude related medical reasons, and one was never healthy at high altitude due to non-altitude related persistent gastrointestinal illness. Thus, 21 subjects (12 males and nine females, average age 20.8 yrs, range 19–23 yrs) constitute the AltitudeOmics group of subjects included in this and subsequent reports (Table 1).

**Table 1:** General Subject Characteristics.

ID	Gender	Age (years)	HT (cm)	WT (kg)	BMI (kg/m^2^)
001	M	22	184.2	80.8	23.8
002	M	22	181.6	65.4	19.8
003	F	21	166.4	54.3	19.6
004	M	21	181.6	70.7	21.4
005	F	21	160.0	53.2	20.7
006	M	19	170.2	68.1	23.5
007	M	21	184.2	73.3	21.6
010	F	19	163.8	67.6	25.1
011	F	21	169.5	68.0	23.6
012	M	20	181.6	82.4	24.9
013	M	23	182.9	77.0	23.0
014	M	21	186.7	85.4	24.4
015	F	22	168.3	56.7	20.0
017	F	23	174.0	69.9	23.0
018	M	21	180.3	79.9	24.5
019	F	19	176.5	68.0	21.8
020	F	19	165.7	62.2	22.6
021	M	20	182.9	68.9	20.6
022	M	23	180.3	73.8	22.6
023	M	20	179.1	77.8	24.2
025	F	19	172.1	60.9	20.5
Mean	12M/9F	20.8	175.8	69.7	22.4
SD		1.4	7.9	9.0	1.8

Height (HT); Weight (WT); Body Mass Index (BMI).

#### Timeline

**Figure 1:**
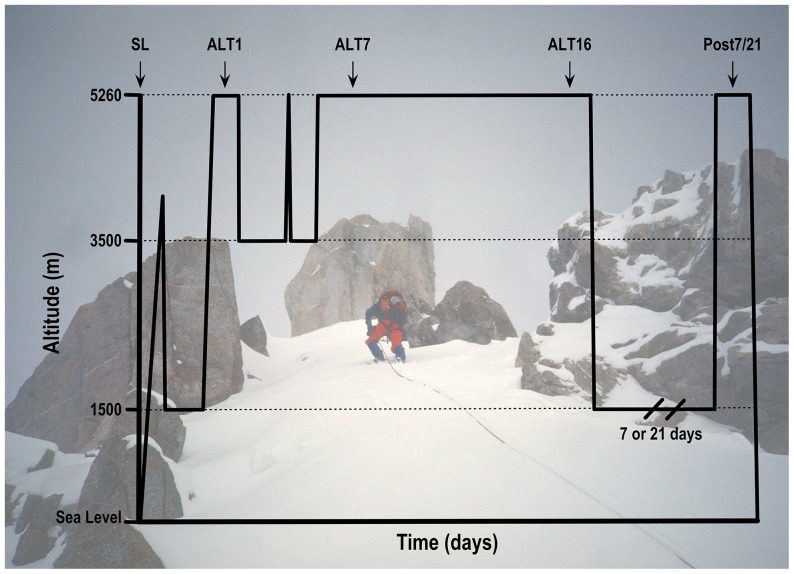
Timeline for AltitudeOmics Studies. Each subject completed this study timeline, with n = 14 staying at low altitude for POST7 and n = 7 staying at low altitude for POST21. Subjects flew from the USA to Bolivia aboard commercial aircraft with no recording of barometric pressure during the flight; the profile for travel in the figure is therefore approximate.

Each subject was studied near sea level (SL) (130 m, average P_B_ = 749 mmHg, Figure 1), and over three study periods at Mt Chacaltaya, Bolivia; 5260 m; average P_B_ = 406 mmHg); on the first/second and sixteenth/seventeenth days at 5260 m (ALT1, ALT16), and again upon reascent to 5260 m, after either seven (n = 14) or 21 (n = 7) days at low altitude (POST7 or POST21). Baseline studies at SL, including laboratory (physiologic and OMICS) and field (3.2-km uphill run) tests, were conducted over a two-week period in Eugene, OR, USA. Approximately one month after the SL studies, subjects traveled to Bolivia in pairs on successive days. Upon arrival at El Alto (4050 m) after an overnight flight, subjects immediately descended to Coroico, Bolivia (1525 m; P_B_ = 639 mmHg). Subjects rested for 48 hrs in Coroico to limit the effects of jet lag and were then driven over three hrs to 5260 m. To provide an acute change in inspired PO_2_ from 1525 m to 5260 m, subjects breathed supplemental oxygen (2 L/min, nasal cannula or mask) during the drive. On arrival at 5260 m, the first subject immediately began the experimental protocol described below. The second subject rested while continuing to breathe supplemental oxygen for ∼ two hrs until the first subject had completed the arterial/venous catheterization and cognitive testing portion of the protocol. Then the second subject began the protocol as described for the first subject. Two subjects were studied per day for ALT1, ALT16, POST7, and POST21. After completing laboratory testing and AMS scoring on ALT1, subjects slept overnight on supplemental oxygen to minimize the risk of developing severe high-altitude illness. The next morning, subjects completed a 3.2-km uphill run (305 m elevation gain) before descending by car to La Paz, Bolivia (3800 m; average P_B_ = 487 mmHg) to continue acclimatizing at a lower altitude over three nights (ALT2-ALT4). On ALT4 subjects visited 5260 m for four to six hrs. On ALT5, they returned to 5260 m, where they remained for an additional 13 days. On ALT16/17 subjects were tested, as on ALT1/2 prior to descending by car to 1525 m. To test physiological retention of acclimatization after living for seven (n = 14) or 21 (n = 7) days at low altitude (1525 m), subjects returned to 5260 m by car, as they did on ALT1 but this time without supplemental oxygen, and completed the POST7/21 testing (detailed below). After completion of a 3.2-km uphill run on POST7/21, the subjects returned home. Assignment to POST7 or POST21 was determined by each subject based on their desire to stay in the field an extra seven or 21 days. While in Bolivia, subjects were housed and fed as a group. Meals and snacks were kept similar to the subjects' typical *ad libitum* diet. Subjects were instructed to ingest at least three liters of water each day and to remain physically active.

#### Experimental Protocol

Testing progressed in the following general order: 1) radial artery and antecubital vein catheterization; 2) 30-min supine rest, followed by cognitive function testing; 3) measurement of resting (seated) arterial blood gases and hemoglobin concentration, and blood draw for OMICS samples; 4) cycle ergometry exercise testing; 5) AMS symptom scoring; and, on a separate day, 6) a 3.2-km uphill run. In addition to the studies presented here, within the framework of AltitudeOmics and reported separately, we also assessed cerebral blood flow[6] and cerebral autoregulation [7]; chemical control of breathing [8]; total hemoglobin mass and blood volume compartments; peripherally [9] and centrally [10] derived measures of exercise-induced fatigue; blood flow through intracardiac shunt (patent foramen ovale) and intrapulmonary arteriovenous anastomoses; and OMICS responses (transcriptomics, epigenomics, metabolomics, and proteomics).

### Procedures

#### Anthropometry

Height (cm) was measured at SL only. Body mass (kg) was recorded at SL, ALT1, ALT16, and POST7/21 using the same scale (Seca 770, Hanover, MD, USA), with the subject wearing light underwear and no shoes.

### Arterial Blood Gases and Hemoglobin

Under local anesthesia (2% lidocaine) a 20–22 G radial artery catheter (Models RA-04122/RA-04020 Arrow International, Reading, PA, USA) was placed for the duration of experiments conducted at SL, ALT1/16, and POST7/21. Arterial blood samples were drawn anaerobically and immediately analyzed in duplicate for PaO_2_, PaCO_2_, pH (Siemens RAPIDLab 248, Erlangen, Germany), [Hb] and SaO_2_ (Radiometer OSM3, Copenhagen, Denmark). Core temperature was measured using an ingestible temperature-sensing pill (CorTemp HQInc, Palmetto, FL, USA)[11], [12]. Blood gases were corrected for core temperature [11], [12]. CaO_2_ (mL/dL) was calculated as: CaO_2_ = 1.39 * [Hb] * (SaO_2_/100)+(PaO_2_ * 0.003). The Hill equation was used to calculate P50 [13]. Resting arterial blood samples were taken following 10 min of seated rest at SL, ALT1, ALT16, and POST7/21.

### Acute Mountain Sickness

The severity of AMS symptoms was assessed using the Lake Louise Questionnaire (LLQ), which includes a self-reported assessment of AMS symptoms (headache, fatigue, gastrointestinal discomfort, and dizziness) and the shortened Environmental Symptom Questionnaire (AMS-C). Total LLQ scores that included headache and were ≥3 or ≥6 (out of a possible total of 12) were diagnostic of moderate or severe AMS, respectively. Quality of sleep was not included in the total LLQ score because nights prior to ALT1 and POST7/21 were spent at low altitude. Recently, in our laboratory, we have published LLQ without using the sleep question, with no change in sensitivity in identifying AMS [14], [15]. AMS-C is a self-reported 11-question inventory from which a score ≥ 0.7 is considered indicative of AMS [16]. AMS symptoms were assessed at SL, ALT1 (in the evening, 10–12 hrs after arrival), ALT16, and POST7/21 (time-matched to ALT1).

### Cognitive Function

The Defense Automated Neurobehavioral Assessment (DANA) was used to assess neurocognitive function. DANA is a neurocognitive assessment tool that includes a library of open-source, standardized, cognitive and psychological assessments [17]. Using a handheld computer, the following nine cognitive function tests were administered: 1) Simple Reaction Time-1 (measured at the beginning of neurocognitive testing to gain an understanding of pure visual-motor response); 2) Simple Reaction Time-2, repeated at the end of neurocognitive testing to assess diminished reserve of cognitive effort on reaction time; 3) Procedural Reaction Time, a measure of choice reaction time and accuracy; 4) Go-No-Go, a measure of speed, accuracy and impulsivity; 5) Code Substitution—Simultaneous, a measure of visual scanning and attention, learning, and immediate recall of digit-symbol pairings; 6) Code Substitution—Delayed Recall, a measure of short-term memory for digit-symbol pairings; 7) Spatial Discrimination, a measure of visuospatial analytic ability; 8) Match to Sample, an assessment of attention and memory for visuospatial discrimination; and 9) Sternberg's Memory Search, a measure of working memory for letters. Neurocognitive tests were administered before and after the expedition at SL and once each at ALT1, ALT16 and POST7/21. Repeat cognitive function tests at SL were similar (p>0.5) and thus were combined to give one SL score for comparison to changes in cognitive function at 5260 m. Mean throughput, a measure of mental efficiency, is calculated as the mean number of correct responses for each test made within one min [18] and is the outcome variable reported for all cognitive function variables.

### Exercise

*Laboratory exercise testing*. Incremental exercise tests to maximal exertion on an electrically-braked cycle ergometer (Velotron Elite, Racermate, Seattle, WA, USA) were used to assess peak aerobic power. Subjects completed three-min stages at 70, 100, 130 and 160 Watts, followed by 15 Watts/min increments until they could no longer maintain pedaling at > 50 rpm. Peak aerobic power (Watts) was calculated as: work rate of last stage completed + [(work rate increment) * (time into final stage/duration of stage in seconds)] [19]. Exercise tests were performed at SL, ALT1, and ALT16, but not at POST7/21 due to logistical issues.

*Field exercise testing*. Subjects completed a timed 3.2-km uphill run as fast as possible, on unpaved roads, with an identical elevation gain of 305 m. Tests were performed at SL at least 48 hrs before the laboratory tests and in the morning after an overnight stay on ALT1, ALT16 and POST7/21. Performance was expressed as mean running speed in m/s.

### Data Analysis

As expected, preliminary analyses revealed higher CaO_2_ for males as a result of higher [Hb], across the study (p<0.01 vs. females); however, since the sex vs. time interaction was not significant (p>0.05) male and female data were pooled for all subsequent analyses. For physiological variables, paired t-tests, with Bonferroni correction for multiple testing, were completed for comparisons among time points. LLQ, AMS-C scores and cognitive function tests were evaluated by the Wilcoxon signed rank test. The Spearman rank order and Pearson product moment correlations were run to evaluate associations between changes in arterial blood gases and [Hb] and changes in AMS symptoms, cognitive function, and physical performance across time. Due to transportation delays and the technical challenges inherent to field studies, not all procedures were completed on all subjects at Mt. Chacaltaya (see Tables S1, S2, S3, S4, S5 for respective sample sizes). Overall, most subjects completed most tests, with 88% of arterial blood gas and hematology measurements, 100% completion of AMS and cognitive function tests, and 95% for the 3.2-km uphill run. For all parametric statistical comparisons, p<0.01 (Bonferroni correction of 0.05/5) was considered significant, with p<0.01 for Wilcoxon signed rank test results considered significant. Individual data for all responses reported here are presented in Tables S1, S2, S3, S4, S5. Data in the text are presented as means ± standard deviation.

## Results

### Anthropometry

Height and body mass at SL are presented in Table 1. Body mass was unchanged from SL to ALT1 (p = NS), then dropped by 2.6±1.6 kg (p<0.01) from ALT1 to ALT16; it showed no significant change thereafter (Table S1).

### Arterial Blood Gases and Hemoglobin

**Figure 2:**
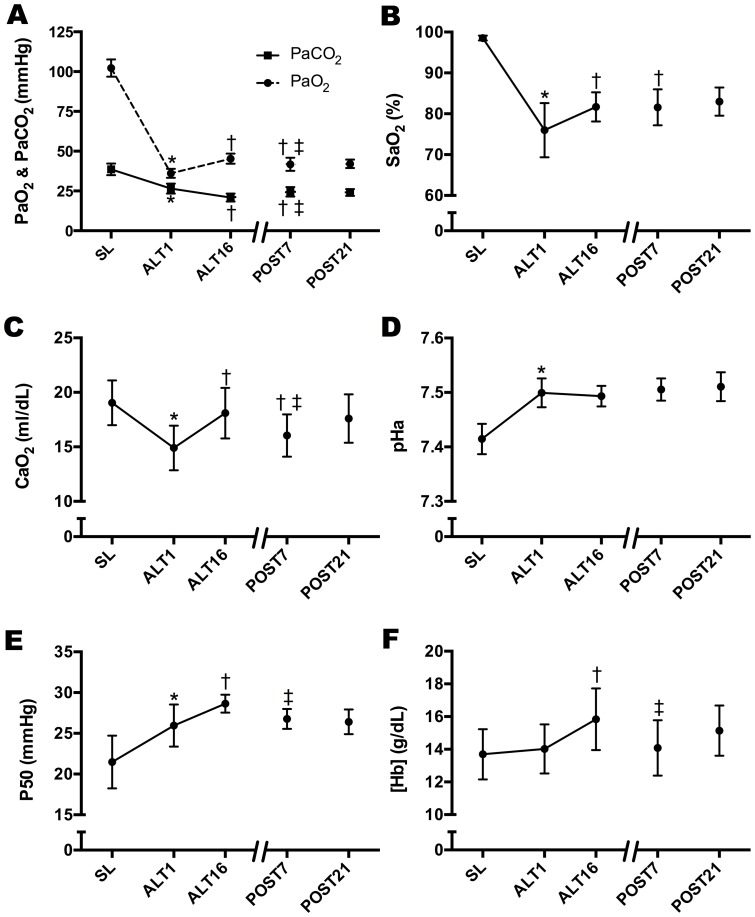
Arterial Blood Gases and [Hb] During Acclimatization and Upon Reascent. Resting indices of ventilatory and hematological acclimatization at SL, ALT1, ALT16, and POST7/21 demonstrating acclimatization after 16 days at a constant altitude and the degree of retention in these variables. *Significantly different vs. SL (p<0.01); ^†^ significantly different than ALT1 (p<0.01); ^‡^significantly different than ALT16 (p<0.01).

PaO_2_, PaCO_2_, SaO_2_, and CaO_2_ were reduced with acute exposure to 5260 m (SL to ALT1, p<0.01; Figure 2, panels A–C, Table S2), while pH and P50 increased (p<0.01, Figure 2, panels D and E) and [Hb] was unchanged (p = NS, Figure 2, panel F). PaO_2_, SaO_2_, CaO_2_, P50, and [Hb] all increased from ALT1 to ALT16, while PaCO_2_ continued to fall (p<0.01, all comparisons) and pH was unchanged (p = NS; Figure 2). SaO_2_ at POST7 was maintained at ALT16 levels. In contrast, PaO_2_, CaO_2_, P50, and [Hb] at POST7 decreased from ALT16 (p<0.01) and approached ALT1 values. PaCO_2_ rose at POST7 from ALT16 values and was significantly different from both ALT1 and ALT16 (p<0.01). Since subjects studied at POST21 had incomplete arterial blood gas data at all time points but SL; those data are qualitatively discussed, but data in the text and figures are at all time points for the POST7 group only. The pattern of change from ALT16 to POST21 was similar to that seen from ALT16 to POST7 for PaO_2_, PaCO_2_, SaO_2_, CaO_2_, pH, and [Hb], suggesting possible retention of acclimatized values for SaO_2_, but less so for PaO_2_, PaCO_2_, CaO_2_, P50, pH, and [Hb].

### Acute Mountain Sickness

**Figure 3:**
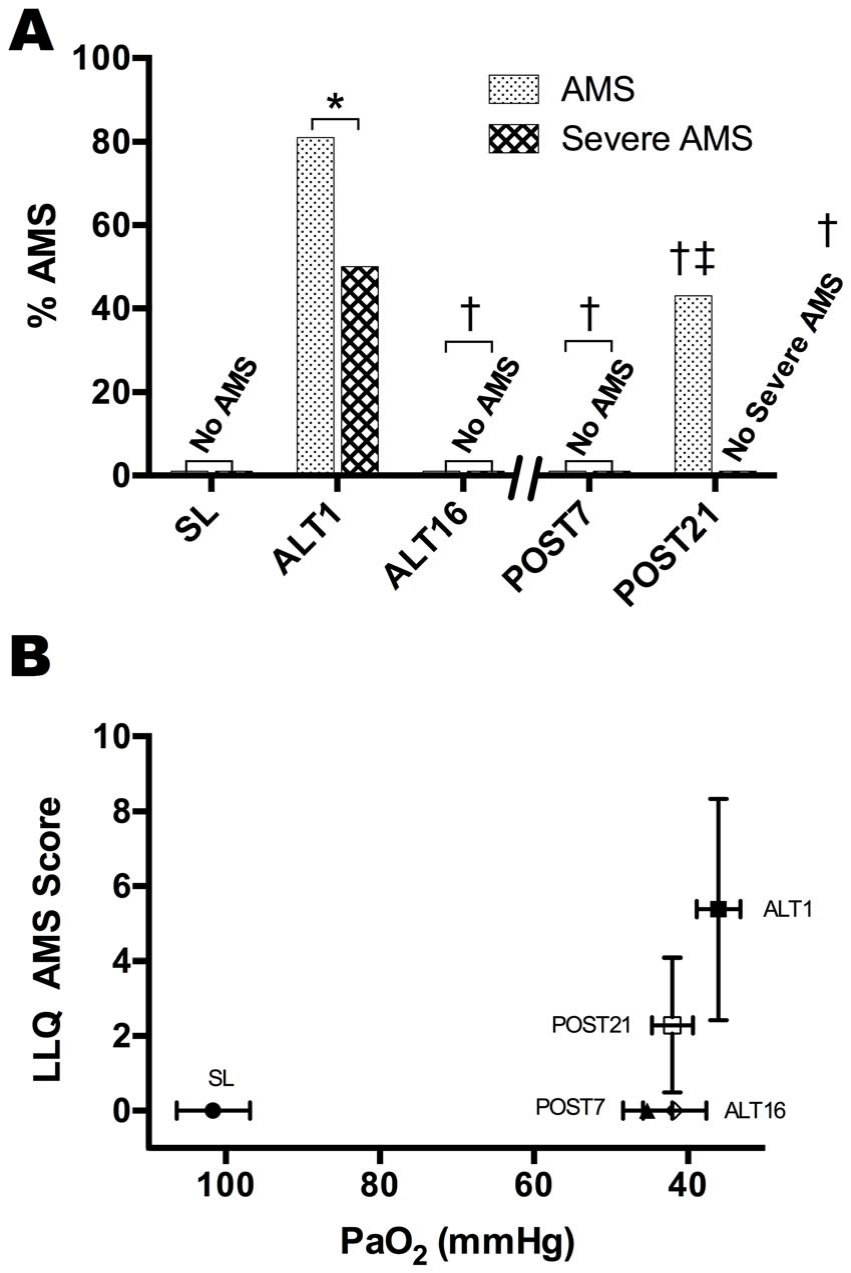
Development of Acute Mountain Sickness, Its Resolution with Acclimatization And Prevention Upon Reascent. Percentage of subjects reporting moderate to severe AMS based on LLQ scores of ≥3, or ≥6, respectively. (A) Symptoms of AMS at ALT1 were alleviated at ALT16 and were largely absent with reascent on POST7/21. (B) Mean PaO_2_ and median LL AMS scores reveal no relationship of hypoxemia to AMS. *Significantly different than SL (p<0.01); ^†^significantly different than ALT1 (p<0.01); ^‡^significantly different than ALT16 (p<0.01).

LLQ and AMS-C were highly correlated (R^2^ = 0.72, p<0.001) and identified the same subjects as AMS positive at ALT1; for brevity, only the LLQ score is discussed (see Table S3). Eighty-one percent (17/21) of subjects had AMS (LLQ≥3; p<0.01 vs. SL) on the evening of their first night at 5260 m; of those with AMS nearly half had severe AMS (LLQ≥6; p<0.01vs. SL; Figure 3A). AMS completely resolved in all subjects as acclimatization progressed from ALT1 to ALT16. Upon reascent at POST7 subjects remained free from AMS. On POST21, 3/7 of subjects again developed AMS scores≥3 (p = NS vs. ALT16), but none reported severe AMS. Nobody exhibited HAPE or HACE.

### Cognitive Function

**Figure 4:**
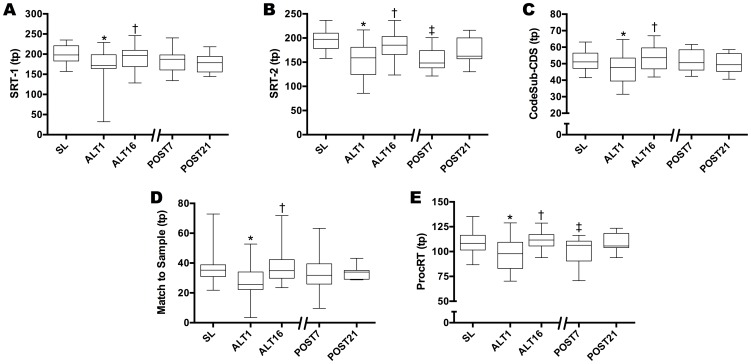
Neurocognitive Function During Acclimatization and Upon Reascent. Five tests of cognitive function revealed marked decrements in performance from SL to ALT1, and improvement back to sea level values by ALT16. Code Substitution—Simultaneous and Match to Sample retained levels found at ALT16 on POST7, while Simple Reaction Time-1, Simple Reaction Time-2, and Procedural Reaction Time essentially reflected a loss of during acclimatization upon reascent at POST7. None of the cognitive function tests showed any retention of acclimatization at POST21. (tp  =  throughput  =  mean number of correct responses made within one min). *Significantly different than SL (p<0.01); ^†^significantly different vs. ALT1 (p<0.01); ^‡^significantly different vs. ALT16 (p<0.01).

Repeat tests at sea level pre-post expedition showed no major differences between individuals or group values (p>0.5) and were thus averaged to provide a more robust SL value (Table S4a–c). Five of nine neurocognitive tests showed marked decrements from SL to ALT1 (Simple Reaction Time-1, Simple Reaction Time-2, Code Substitution—Simultaneous, Match to Sample and Procedural Reaction Time, p<0.01, Figure 4); no change from SL to ALT1 was seen for Code Substitution—Delayed Recall, Spatial Discrimination, Go-No-Go, and Memory Search (p>0.05) (Table S4a–c). Subsequent analyses focused on the five tests that showed a change with acute hypoxia. Performance improved on Simple Reaction Time-1, Simple Reaction Time-2, Code Substitution—Simultaneous, Match to Sample, and Procedural Reaction Time as acclimatization progressed from ALT1 to ALT16 (p<0.01, Figure 4). At POST7, Code Substitution—Simultaneous and Match to Sample showed retention of acclimatization compared to ALT16 (p<0.01, Figure 4, panels C and D), with loss of acclimatization evident for Simple Reaction Time-2, Procedural Reaction Time (p<0.01, Figure 4, panel B and E), and a trend to loss of acclimatization noted for Simple Reaction Time-1 (p>0.01<0.05, Figure 4, panel A). No cognitive function tests showed retention of acclimatization at POST21.

### Exercise

*Laboratory exercise testing*. Peak oxygen uptake at SL was 3.4±0.8 l/min and fell by 29±11% to 2.3±0.6 l/min at ALT1 (p<0.01), with no change observed from ALT1 to ALT16 (p = NS) (See Table S5). Peak power output at SL was 265±57 W; it fell by 34±7% to 171±40 W at ALT1 (p<0.01), and like peak oxygen uptake, it did not improve with acclimatization. Changes in resting arterial oxygenation and [Hb] from SL to ALT1 to ALT16 were not correlated with peak oxygen uptake (p = NS).

**Figure 5:**
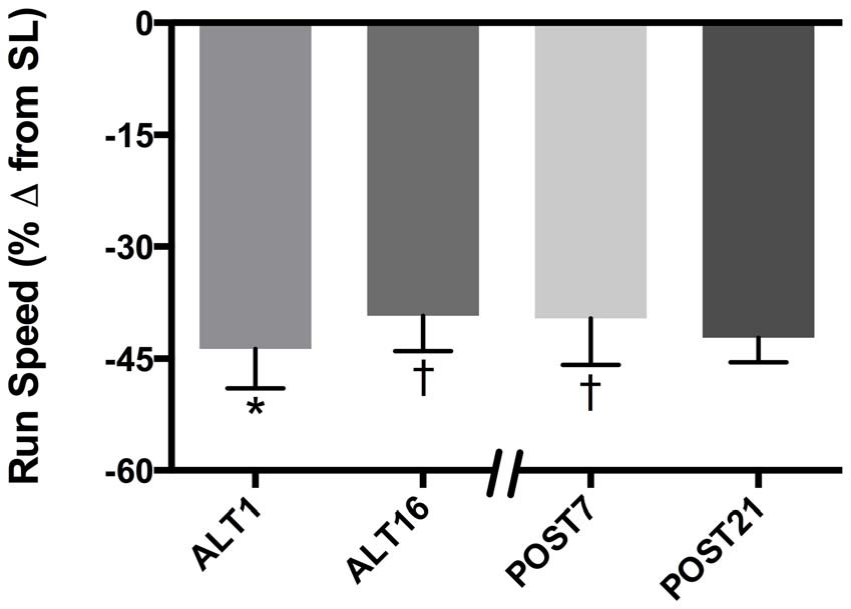
Field Exercise Testing During Acclimatization and Upon Reascent. Uphill running speed plotted as percent change from sea level improved from ALT1 to ALT16 and was retained at POST7, with a trend to retention at POST21. *Significantly different vs. SL (p<0.01); ^†^significantly different vs. ALT1 (p<0.01).

*Field exercise testing*. Running speed was 44±5% slower at ALT1 compared to SL (p<0.01; Figure 5). Running speed improved 8±8% from ALT1 to ALT16 (p<0.01) and was maintained at POST7 (p = NS). Subjects maintained acclimatized (ALT16) running speed at POST7 despite 13% lower resting [Hb] and CaO_2_. After 21 days at low altitude, running speed tended to be slower than at ALT16 (p = 0.06) and was not significantly different from ALT1 (p = NS), suggesting a partial loss of acclimatization in running speed by POST21.

### Relationship of AMS, Cognitive Function and Exercise Performance to Arterial Oxygenation and [Hb]

During acclimatization AMS, cognitive function, and exercise performance improved, and for AMS and exercise those improvements were retained upon reascent, with only some tests of cognitive function showing retention of acclimatization. The changes that occurred during acclimatization and upon reascent in PaO_2_, PaCO_2_, SaO_2_, CaO_2_, P50, pH, and [Hb] were not related on an individual (all correlations r<0.5) or group basis (all comparisons p>0.1) to AMS, cognitive function, or exercise responses. However, the pattern of change with acclimatization in PaO_2_, PaCO_2_, SaO_2_, CaO_2_, P50, pH and [Hb] matches the pattern of change for AMS, cognitive function, and exercise performance, suggesting an underlying but complex relationship between oxygenation and other aspects of acclimatization.

## Discussion

In this paper, we have presented four aspects of altitude acclimatization through a 16-day initial exposure to 5260 m, and upon reascent to the same altitude after either seven or 21 days at low altitude. We found, as have others before us [20]–[30], elevated arterial oxygenation and [Hb], resolution of symptoms of acute mountain sickness and increased exercise performance after 16 days residence at 5260 m. We also report improvements in measures of cognitive performance that we believe represent a novel and important additional indicator of acclimatization. Most intriguing was finding that after descending to low altitude for one or three weeks, physiological evidence of acclimatization persisted upon returning to 5260 m, as manifest by less AMS, retention of improved exercise performance, and to some extent cognitive performance.

### Physiology of Acclimatization

The elevations in arterial oxygenation and [Hb] from ALT1 to ALT16 were similar to those measured in individuals acclimatized for at least 10 days at altitudes ranging from 3800 m to 5260 m [20], [26], [29], [30]. For example, Lundby et al. reported that [Hb] and CaO_2_ increased markedly from SL to two weeks at 4100 m, but did not rise further at eight weeks [26]. While similar data do not exist for the rise in PaO_2_ and fall in PaCO_2_ with ventilatory acclimatization at two and eight weeks at a fixed high altitude, Wagner et al. reported after nine weeks at 5260 m a PaO_2_ of 50±1 mmHg and a PaCO_2_ of 21±0.9, values similar to PaO_2_ (45±3) and PaCO_2_ (21±3) in the present study after 16 days at 5260 m [30]. Thus, it seems that≥14 days at 4000 m to 5000 m results in significant acclimatization, and that this duration of exposure can be effective to test acclimatization and its subsequent retention [30].

Sixteen days of acclimatization at 5260 m was effective in reducing the incidence of AMS from 81% in our subjects upon acute exposure to 0% at ALT16, a finding consistent with existing literature [23], [24], [28]. These findings suggest a new experimental approach to unraveling the pathophysiology of AMS. To our knowledge, no pathophysiological studies of AMS have taken advantage of the complete protection from AMS conferred by acclimatization by comparing individuals upon acute ascent to when they are acclimatized, or upon reascent when presumably the factors that protect from AMS will stand out from other factors that are epiphenomena to the acclimatization process but not key to AMS prevention.

This is the first report of complete recovery of cognitive function to sea level values after acclimatization to high altitude, supporting the idea that cognitive function is an important outcome of acclimatization. DANA tests have negligible practice effects (other than spatial discrimination, which asymptotes after the second administration) [17]. This was evident in the current study, as no significant differences were detected between DANA measures on pre- and post-expedition SL tests. We found that the five tests showing impairment in acute hypoxia all returned to SL values by ALT16 (p<0.01, Figure 4). Barcroft et al. reported anecdotal impairment in cognitive function during acclimatization, but lacked any quantitative evidence [31]. Other studies have reported effects on cognitive function in acute hypoxia [32]–[36] during experiments and expeditions where the barometric pressure and environmental conditions were different at each testing point, such as occurs during a climbing expedition [37]–[39], and one has speculated about the recovery of cognitive function with acclimatization [40]. However, none of those studies have shown, as in the present study, that when subjects are studied at the same altitude over the course of acclimatization that cognitive function improves to sea level values. DANA tests speed and accuracy in measures that assess attention, simple discrimination, and immediate and incidental memory. Although these measures offer an indication of working memory, they do not assess complex problem-solving and decision-making aspects of executive functioning, which may be especially relevant for people working at high altitudes. Understanding the mechanism for the marked resolution of the initial decrement in cognitive performance that occurs in acute hypoxia has potential impact [41] for anyone visiting, living, or working at high altitudes where impaired cognitive dysfunction is a major challenge [37], [38], [42].

Our findings for submaximal exercise performance are consistent with other reports showing improvements during acclimatization [22], [25], [27], [43] with no change in peak oxygen consumption [2], [22], [26], [44]–[51]. However, in retrospect, we question the practical relevance of these all-out efforts, as most work or recreational activities at high altitude are not performed to exhaustion or as fast as possible. For example, mountaineers try to preserve energy to sustain efforts across multiple days and might actually put themselves at risk of serious harm, or death, if they truly reached the point of exhaustion. Their ability to cover more ground faster while preserving a functional reserve is a hallmark of acclimatization supported by anecdotal accounts [52], [53]. To the best of our knowledge, only one study before the present report has objectively measured this type of submaximal performance [43]. The physiology behind the improvement in sustained, self-regulated submaximal performance at altitude remains unexplored [2], [22], [26], [43]–[52].

### Physiological Retention of Acclimatization: Arterial Blood Gases and Hemoglobin

At POST7/21, PaO_2_ and PaCO_2_ values ranged between ALT16 and ALT1 values, indicating partial retention of ventilatory acclimatization. In contrast, SaO_2_ and pH remained near ALT16 acclimatized levels on POST7/21. We calculated a decreased P50 from ALT16 to POST7/21, suggesting a left shift in the oxyhemoglobin dissociation curve upon reascent as a possible explanation for the retention of acclimatized values for SaO_2_ at POST7/21 [2]. These findings are compatible with one previous study showing partial retention of ventilatory acclimatization using noninvasive indices of oxygenation and end tidal CO_2_ levels after eight days at low altitude [2], [54]. The drop in [Hb] from ALT16 to POST7/21 may be due to selective destruction of the youngest circulating red cells (neocytolysis) upon return to low altitude [55]–[58], or potentially an increase in plasma volume [59].

### Physiological Retention of Acclimatization: Acute Mountain Sickness

Our findings on AMS upon reascent extend the work of others conducted at lower altitudes in demonstrating that previous altitude acclimatization confers some protection from AMS [3], [4], [60]. The marked efficacy of acclimatization to prevent severe AMS is underscored by comparison to results from clinical trials where acetazolamide only reduced the risk of severe AMS by 44% [61], compared to 100% for acclimatization in our study. Exactly how acclimatization prevents AMS and other high-altitude illnesses upon reascent is unclear.

AMS is clearly triggered by hypoxemia, but once the processes that cause AMS are initiated, the relationship with PaO_2_, SaO_2_, and CaO_2_ is less clear. This is reflected in Figure 3B where AMS scores are highest when PaO_2_ is lowest at ALT1, but when at POST7 and ALT16, when PaO_2_ levels are only a few mmHg higher than ALT1 values, AMS is absent. Additionally, at POST7, when AMS is absent in all 14 subjects, CaO_2_ levels are much lower than at ALT16, suggesting a limited role for CaO_2_ in the protection from AMS observed upon reascent. One explanation may be that the absolute value of PaO_2_, SaO_2_, or CaO_2_ is not the critical factor, but rather that acute hypoxia sets in motion the physiological alterations leading to AMS. In other words, perhaps an individual threshold exists that triggers AMS when crossed [62]. Unraveling how this occurs may lead to advances in the understanding of the pathophysiology of high-altitude illnesses.

### Physiological Retention of Acclimatization: Cognitive Function

Cognitive function stands out as a key feature of acclimatization to hypoxia that is not completely retained at acclimatized levels upon reascent. The tests that showed retention of acclimatization at POST7 (Code Substitution—Simultaneous and Match to Sample) commonly reflect changes in short-term memory. The tests of reaction time (Simple Reaction Time-1, Simple Reaction Time-2, Procedural Reaction Time) essentially returned to ALT1 values by POST7, indicating a loss of the improvement in reaction time seen with acclimatization. Short-term memory and reaction time appear to represent distinct processes that respond differently to the changes in arterial blood gases and [Hb] from ALT16 to POST7. Understanding the mechanisms responsible for acclimatization retention or its loss could lead to new insights into the links between brain oxygenation and cognitive function for persons at high altitudes.

### Physiological Retention of Acclimatization: Exercise

The retention of exercise performance for at least seven days, with partial retention after 21 days spent at low altitude, has important implications for everyone living, visiting, or working at high altitudes. At POST7, and to a lesser extent at POST21, subjects essentially matched their acclimatized running performance. This is the first report of retention of acclimatized exercise performance upon reascent after de-acclimatizing at low altitude. As far as we know, only one other study attempted to measure retention of acclimatized endurance exercise performance [2], but that study showed no improvement in endurance exercise performance with acclimatization, likely due to a small sample size (n = 6), thus rendering testing of retention impossible. As noted above, all studies [22], [25], [27], [43] but one [2] have shown improvement in submaximal endurance capacity with acclimatization. The retention of exercise performance shown at POST7 occurred despite significant reductions in resting [Hb] and CaO_2_. These findings are contrary to those reporting a direct positive effect of CaO_2_ on exercise performance at lower altitudes [25], [63], but agree with those reporting little effect of CaO_2_ on exercise performance at higher altitudes (>3500 m) [64]–[66]. If the improvement of exercise performance with acclimatization and its retention upon reascent is not directly related to CaO_2_, then other factors must be at play. One possibility is that mechanisms other than oxygen delivery could boost oxygen transport and thus exercise performance during acclimatization and upon reascent, such as elevated circulating blood levels of vasodilatory substances (e.g., nitric oxide [67] or adenosine [68]) or other, as yet unknown, processes. Discovering the mechanisms responsible for improving exercise performance with acclimatization and its retention after acclimatization has potential relevance to exercise tolerance in anyone exposed to hypoxia.

### Physiological Mechanisms Explaining Acclimatization and its Retention

Acclimatization transforms a lowlander into someone who is protected from high-altitude illness, has improved cognitive function, and has better exercise performance at 5260 m. In the present study, acclimatization-induced improvements in AMS symptoms, cognitive function and exercise performance appear to follow the time course of ventilatory and hematological acclimatization. But after extensive analysis, no case was found where the degree of improvement in AMS symptoms, cognitive function, and exercise performance was significantly directly correlated to measured indices of arterial oxygenation and [Hb]. Further, arterial oxygenation and [Hb] were poorly correlated with the benefits of acclimatization that persisted upon reascent. Though not well known, Luft et al. reported on the retention of acclimatization based on studies conducted in hypobaric chambers on climbers returning from Nanga Parbat in 1938 [69]. The measurement of retention was tolerance to very high altitudes (>8000 m) measured, in part, by deterioration of handwriting. They noted that neither the hemoglobin concentration nor the erythrocyte count were responsible for the persistence of acclimatization. While we acknowledge the inherent limitations of correlational analyses, the disconnection between ventilatory and hematological acclimatization and physiological function suggests that additional mechanisms are involved in acclimatization and its retention. These might include physiological responses that we did not measure, or molecular and cellular responses in a specific tissue such as brain that cannot be easily measured in humans. In subsequent reports we will pursue a linkage between the OMICS responses and the physiological adjustments described here to explore the mechanisms underlying acclimatization to high altitude and its retention.

### Limitations

Several limitations in the study design and execution should be considered. This study was completed in the field, in a foreign country, and with many uncontrolled variables. The rationale for this approach over a trial in a hypobaric environment where many more variables could be controlled was that such a large study could not be completed for a reasonable cost and in a reasonable time-frame in a hypobaric chamber. Operation Everest II studied six-to-eight subjects during a 40-day simulated ascent of Mt Everest. Though many of the time points from Operation Everest II had data from only four to six subjects, many important observations were made from these experiments [29], [44], [70]–[73]. But to have sufficient statistical power to combine the OMICS and physiological studies, much larger sample sizes are needed. As far as the authors know, there is one hypobaric chamber in the world large enough to accommodate 21 subjects at a time, located in Glasgow, Scotland. While we acknowledge the field design as a limitation, we believe this study makes an important contribution to understanding acclimatization that can point to future studies with smaller samples and more focused experimental questions in controlled hypobaric chamber conditions.

This study was limited to 16 days of acclimatization. While this was sufficient time to see marked changes in the variables measured, it is unclear if longer exposure would have resulted in further improvements in acclimatization or better retention of acclimatization upon reascent. Also, due to logistical and financial constraints and to avoid areas of high malarial risk, subjects did not descend all the way to sea level between exposures. However, this may not be a major concern, since our results are consistent with other studies reporting protection from AMS after acclimatization [3], [4], [60]. Only Lyons et al. [3] reported data from a controlled study of acclimatizing individuals; others used epidemiological observations suggesting AMS protection from acclimatization [4], [60]. Also, we made no measurements at low altitude prior to reascent, so a question remains as to how much of the reascent responses were present at low altitude such as hyperventilation, resulting in low PaCO_2_, versus how much was nascent at low altitude but was rapidly triggered on re-ascent.

An additional concern is that subjects may have de-trained over the 16 days at high altitude, since they were unable to completely maintain their regular exercise regimen. When back at low altitude, subjects resumed their habitual levels of physical activity, potentially restoring some fitness and confounding our measures of exercise performance. Also, changes in total and lean body mass across the study may have affected physical performance [74], but since changes in body composition and training status are inherent to life at high altitude, we feel our results have strong practical relevance.

Finally, the AltitudeOmics project encompasses an extensive suite of physiological and OMICS measurements, and, in its entirety, produced more than 60 million individual data points. Consequently, the data has been partitioned into discrete papers with the ultimate goal of a series of publications that are individually robust and as comprehensive as possible. The physiological parameters included in this paper have historically been used to describe acclimatization, and thus were deemed appropriate as a bridge between past studies and the novel discoveries from AltitudeOmics. Further publications will explore the process of acclimatization by utilizing additional OMICS and physiological data whose inclusion excessively widened the scope of the current paper.

### Conclusion

In this study of acclimatization to a very high altitude, we found improvements in key variables after 16 days that describe an acclimatized phenotype by partial acclimatization for arterial oxygenation and [Hb], absence of high-altitude illness, improved cognition and exercise performance. Another intriguing observation is that after descending to low altitude for one or three weeks, evidence of acclimatization persists, as manifested by an acclimatized value for SaO_2_, much less severe AMS, maintained exercise performance, and to a lesser extent retention of acclimatized cognitive performance. During the time at low altitude, many of the changes reflecting ventilatory and hematologic adaptation returned to or toward the unacclimatized state at the time reascent measurements were made. In conclusion, this study identifies a phenotype of successful human acclimatization to hypoxia, identifies novel aspects of the retention of the acclimatized phenotype after time at low altitude, and will serve as a foundation for comparing the phenotype of acclimatization with potential mechanistic mediators of acclimatization derived from companion studies of the human transcriptome, epigenome, metabolome, and proteome.

## Supporting Information

Table S1 **Body Composition.** Individual body weight data at SL, ALT1, ALT16, POST7 and POST21 and body fat and lean body mass at SL, ALT1, and ALT16.
(PDF)

Table S2 **Resting Arterial Blood Gases and Hemoglobin Concentration.** Individual resting arterial blood gases and [Hb] data at SL, ALT1, ALT16, POST7 and POST21.
(PDF)

Table S3 **Acute Mountain Sickness Scores for Lake Louise (LLQ) and Environmental Symptom (AMS-C) Questionnaires.** Individual AMS symptom scores and the composite LL and AMS-C scores at SL, ALT1, ALT16, POST7 and POST21.
(PDF)

Table S4 a. Cognitive Function Tests. Individual cognitive function test scores for Simple Reaction Time-1, Simple Reaction Time-2, Code Substitution—Simultaneous, and Code Substitution—Delayed Recall at SL, ALT1, ALT16, POST7 and POST21. b. Cognitive Function Tests. Individual cognitive function test scores for Spatial Discrimination, Go-No-Go, Sternberg's Memory Search, and Matching to Sample at SL, ALT1, ALT16, POST7 and POST21. c. Cognitive Function Tests. Individual cognitive function test score for Procedural Reaction Time at SL, ALT1, ALT16, POST7 and POST21.
(PDF)

Table S5 **Peak Power Output and Submaximal Exercise Performance.** Individual maximal exercise performance and 5-km time to completion data at SL, ALT1, and ALT16 and field exercise testing results at SL, ALT1, ALT16, POST7 and POST21.
(PDF)

## References

[1] Hultgren H (1997) High Altitude Medicine. Standford, CA, USA: Hultgren Publications.

[2] Beidleman BA, Muza SR, Rock PB, Fulco CS, Lyons TP, et al (1997) Exercise responses after altitude acclimatization are retained during reintroduction to altitude. Medicine and Science in Sports and Exercise 29: 1588–1595. 9432091 10.1097/00005768-199712000-00007

[3] Lyons TP, Muza SR, Rock PB, Cymerman A (1995) The effect of altitude pre-acclimatization on acute mountain sickness during reexposure. Aviation, Space, and Environmental Medicine 66: 957–962. 8526832

[4] Wu TY, Ding SQ, Liu JL, Yu MT, Jia JH, et al (2009) Reduced incidence and severity of acute mountain sickness in Qinghai-Tibet railroad construction workers after repeated 7-month exposures despite 5-month low altitude periods. High Altitude Medicine and Biology 10: 221–232. 19775211 10.1089/ham.2009.1012

[5] Knapik J (1989) The Army Physical Fitness Test (APFT): a review of the literature. Military Medicine 154: 326. 2498771

[6] Subudhi AW, Fan JL, Evero O, Bourdillon N, Kayser B, et al. (2013) AltitudeOmics: Effect of ascent and acclimatization to 5260 m on regional cerebral oxygen delivery. Exp Physiol (PMID: 24243839, DOI:10.1113/expphysiol.2013.075184). 24243839

[7] Subudhi AW, Fan JL, Evero O, Bourdillon N, Kayser B, et al. (2013) AltitudeOmics: Cerebral autoregulation during ascent, acclimatization, and re-exposure to high altitude and its relation with acute mountain sickness. J Appl Physiol (1985) (PMID: 24371013; DOI:10.1152/japplphysiol.00880.2013). 24371013

[8] Fan JL, Subudhi AW, Evero O, Bourdillon N, Kayser B, et al. (2013) AltitudeOmics: Enhanced cerebrovascular reactivity and ventilatory response to CO2 with high altitude acclimatisation and re-exposure. J Appl Physiol (1985) (PMID: 24356520, DOI:10.1152/japplphysiol.00704.2013). 24356520

[9] Amann M, Goodall S, Twomey R, Subudhi AW, Lovering AT, et al (2013) AltitudeOmics: On the consequences of high-altitude acclimatization for the development of fatigue during locomotor exercise in humans. J Appl Physiol (1985) 115: 634–642. 23813531 10.1152/japplphysiol.00606.2013 PMC3763067

[10] Goodall S, Twomey R, Amann M, Ross EZ, Lovering AT, et al. (2014) AltitudeOmics: Exercise-induced supraspinal fatigue is attenuated in healthy humans after acclimatisation to high altitude. Acta Physiol (PMID: 24450855, DOI:10.1111/apha.12241). PMC4291113 24450855

[11] Kelman GR (1966) Digital computer subroutine for the conversion of oxygen tension into saturation. Journal of Applied Physiology 21: 1375–1376. 5916678 10.1152/jappl.1966.21.4.1375

[12] Severinghaus JW (1966) Blood gas calculator. Journal of Applied Physiology 21: 1108–1116. 5912737 10.1152/jappl.1966.21.3.1108

[13] Hill AV (1910) The possible effects of the aggregation of the molecules of hemoglobin on its dissociation curves. Proceedings of the Physiological Society 40: : i–vii.

[14] Julian CG, Subudhi AW, Wilson MJ, Dimmen AC, Pecha T, et al (2011) Acute mountain sickness, inflammation, and permeability: new insights from a blood biomarker study. Journal of Applied Physiology 111: 392–399. 21636566 10.1152/japplphysiol.00391.2011 PMC3154693

[15] Subudhi AW, Dimmen AC, Julian CG, Wilson MJ, Panerai RB, et al (2011) Effects of acetazolamide and dexamethasone on cerebral hemodynamics in hypoxia. Journal of Applied Physiology 110: 1219–1225. 21393464 10.1152/japplphysiol.01393.2010 PMC3098658

[16] Sampson JB, Kobrick JL (1980) The environmental symptoms questionnaire: revisions and new field data. Aviation, Space, and Environmental Medicine 51: 872–877. 7417157

[17] Lathan C, Spira JL, Bleiberg J, Vice J, Tsao JW (2013) Defense Automated Neurobehavioral Assessment (DANA)-psychometric properties of a new field-deployable neurocognitive assessment tool. Military Medicine 178: 365–371. 23707818 10.7205/MILMED-D-12-00438

[18] Thorne DR (2006) Throughput: a simple performance index with desirable characteristics. Behavior Research Methods 38: 569–573. 17393825 10.3758/bf03193886

[19] Subudhi AW, Lorenz MC, Fulco CS, Roach RC (2008) Cerebrovascular responses to incremental exercise during hypobaric hypoxia: effect of oxygenation on maximal performance. American Journal of Physiology-Heart & Circulatory Physiology 294: H164–171. 18032522 10.1152/ajpheart.01104.2007

[20] Bebout DE, Story D, Roca J, Hogan MC, Poole DC, et al (1989) Effects of altitude acclimatization on pulmonary gas exchange during exercise. Journal of Applied Physiology 67: 2286–2295. 2606834 10.1152/jappl.1989.67.6.2286

[21] Calbet JAL (2003) Chronic hypoxia increases blood pressure and noradrenaline spillover in healthy humans. Journal of Physiology 551: 379–386. 12844510 10.1113/jphysiol.2003.045112 PMC2343162

[22] Fulco CS, Kambis KW, Friedlander AL, Rock PB, Muza SR, et al (2005) Carbohydrate supplementation improves time-trial cycle performance during energy deficit at 4,300-m altitude. Journal of Applied Physiology 99: 867–876. 15879171 10.1152/japplphysiol.00019.2005

[23] Hackett PH, Rennie D, Levine HD (1976) The incidence, importance, and prophylaxis of acute mountain sickness. Lancet 2: 1149–1155. 62991 10.1016/s0140-6736(76)91677-9

[24] Hackett PH, Roach RC (2001) Current concepts: High-altitude illness. The New England Journal of Medicine 345: 107–114. 11450659 10.1056/NEJM200107123450206

[25] Horstman D, Weiskopf R, Jackson RE (1980) Work capacity during 3-wk sojourn at 4,300 m: effects of relative polycythemia. Journal of Applied Physiology 49: 311–318. 7400014 10.1152/jappl.1980.49.2.311

[26] Lundby C, Calbet JA, van Hall G, Saltin B, Sander M (2004) Pulmonary gas exchange at maximal exercise in Danish lowlanders during 8 wk of acclimatization to 4,100 m and in high-altitude Aymara natives. American Journal of Physiology-Regulatory, Integrative and Comparative Physiology 287: R1202–1208. 10.1152/ajpregu.00725.2003 15191909

[27] Maher JT, Jones LG, Hartley LH (1974) Effects of high-altitude exposure on submaximal endurance capacity of men. Journal of Applied Physiology 37: 895–898. 4436221 10.1152/jappl.1974.37.6.895

[28] Singh I, Khanna PK, Srivastava MC, Lal M, Roy SB, et al (1969) Acute mountain sickness. The New England Journal of Medicine 280: 175–184. 5782719 10.1056/NEJM196901232800402

[29] Wagner PD, Sutton JR, Reeves JT, Cymerman A, Groves BM, et al (1987) Operation Everest II: pulmonary gas exchange during a simulated ascent of Mt. Everest. Journal of Applied Physiology 63: 2348–2359. 3436869 10.1152/jappl.1987.63.6.2348

[30] Wagner PD, Araoz M, Boushel R, Calbet JA, Jessen B, et al (2002) Pulmonary gas exchange and acid-base state at 5,260 m in high-altitude Bolivians and acclimatized lowlanders. Journal of Applied Physiology 92: 1393–1400. 11896002 10.1152/japplphysiol.00093.2001

[31] Barcroft J, Binger CA, Bock AV, Doggart JH, Forbes JS, et al (1923) Observations upon the effect of high altitude on the physiological processes of the human body, carried out in the Peruvian Andes, chiefly at Cerro de Pasco. Philos Trans R Soc Lond Ser B 211: 351–480.

[32] Kida M, Imai A (1993) Cognitive performance and event-related brain potentials under simulated high altitudes. Journal of Applied Physiology 74: 1735–1741. 8514690 10.1152/jappl.1993.74.4.1735

[33] Leifflen D, Poquin D, Savourey G, Barraud PA, Raphel C, et al (1997) Cognitive performance during short acclimation to severe hypoxia. Aviation Space and Environmental Medicine 68: 993–997. 9383498

[34] Pavlicek V, Schirlo C, Nebel A, Regard M, Koller EA, et al (2005) Cognitive and emotional processing at high altitude. Aviation Space and Environmental Medicine 76: 28–33. 15672983

[35] Regard M, Landis T, Casey J, Maggiorini M, Bartsch P, et al (1991) Cognitive changes at high altitude in healthy climbers and in climbers developing acute mountain sickness. Aviation Space and Environmental Medicine 62: 291–295. 2031628

[36] Stamper DA, Kinsman RA, Evans WO (1970) Subjective symptomatology and cognitive performance at high altitude. Perceptual and Motor Skills 31: 247–261. 5454492 10.2466/pms.1970.31.1.247

[37] Cauchy E, Larmignat P, Boussuges A, Le Roux G, Charniot JC, et al (2002) Transient neurological disorders during a simulated ascent of Mount Everest. Aviation Space and Environmental Medicine 73: 1224–1229. 12498553

[38] Hornbein TF, Townes BD, Schoene RB, Sutton JR, Houston CS (1989) The cost to the central nervous system of climbing to extremely high altitude. The New England Journal of Medicine 321: 1714–1719. 2512483 10.1056/NEJM198912213212505

[39] Kennedy RS, Dunlap WP, Banderet LE, Smith MG, Houston CS (1989) Cognitive performance deficits in a simulated climb of Mount Everest: Operation Everest II. Aviation Space and Environmental Medicine 60: 99–104. 2930431

[40] Muza SR, Beidleman BA, Fulco CS (2010) Altitude preexposure recommendations for inducing acclimatization. High Altitude Medicine and Biology 11: 87–92. 20586592 10.1089/ham.2010.1006

[41] Dodd JW, Getov SV, Jones PW (2010) Cognitive function in COPD. European Respiratory Journal 35: 913–922. 20356988 10.1183/09031936.00125109

[42] Gerard AB, McElroy MK, Taylor MJ, Grant I, Powell FL, et al (2000) Six percent oxygen enrichment of room air at simulated 5,000 m altitude improves neuropsychological function. High Altitude Medicine and Biology 1: 51–61. 11258587 10.1089/152702900320685

[43] Latshang TD, Turk AJ, Hess T, Schoch OD, Bosch MM, et al. (2011) Acclimatization improves submaximal exercise economy at 5533 m. Scandinavian Journal of Medicine and Science in Sports. 10.1111/j.1600-0838.2011.01403.x 22093058

[44] Sutton JR, Reeves JT, Wagner PD, Groves BM, Cymerman A, et al (1988) Operation Everest II: oxygen transport during exercise at extreme simulated altitude. Journal of Applied Physiology 64: 1309–1321. 3132445 10.1152/jappl.1988.64.4.1309

[45] Wolfel EE, Groves BM, Brooks GA, Butterfield GE, Mazzeo RS, et al (1991) Oxygen transport during steady-state submaximal exercise in chronic hypoxia. Journal of Applied Physiology 70: 1129–1136. 2032978 10.1152/jappl.1991.70.3.1129

[46] Lundby C, Calbet JA, Sander M, van Hall G, Mazzeo RS, et al (2007) Exercise economy does not change after acclimatization to moderate to very high altitude. Scandinavian Journal of Medicine and Science in Sports 17: 281–291. 17501869 10.1111/j.1600-0838.2006.00530.x

[47] Calbet JAL, Robach P, Lundby C, Boushel R (2008) Is pulmonary gas exchange during exercise in hypoxia impaired with the increase of cardiac output? Applied Physiology, Nutrition, and Metabolism 33: 593–600. 10.1139/H08-010 18461116

[48] Calbet JAL, Boushel R, Rådegran G, Søndergaard H, Wagner PD, et al (2003) Determinants of maximal oxygen uptake in severe acute hypoxia. American Journal of Physiology-Regulatory, Integrative and Comparative Physiology 284: R291–303. 10.1152/ajpregu.00155.2002 12388461

[49] Calbet JA, Radegran G, Boushel R, Sondergaard H, Saltin B, et al (2004) Plasma volume expansion does not increase maximal cardiac output or VO_2_ max in lowlanders acclimatized to altitude. American Journal of Physiology-Heart & Circulatory Physiology 287: H1214–1224. 15142851 10.1152/ajpheart.00840.2003

[50] Calbet JA, Boushel R, Radegran G, Sondergaard H, Wagner PD, et al (2003) Why is VO_2_ max after altitude acclimatization still reduced despite normalization of arterial O_2_ content? American Journal of Physiology-Regulatory, Integrative and Comparative Physiology 284: R304–316. 10.1152/ajpregu.00156.2002 12388462

[51] Fulco CS, Friedlander AL, Muza SR, Rock PB, Robinson S, et al (2002) Energy intake deficit and physical performance at altitude. Aviation, Space, and Environmental Medicine 73: 758–765. 12182215

[52] Houston CS, Harris DE, Zeman EJ (2005) Going Higher: Oxygen, Man and Mountains. Seattle, WA: The Mountaineers Books.

[53] Messner R (1989) The Crystal Horizon. Everest The First Solo Ascent. Seattle: The Mountaineers.

[54] Muza SR, Fulco CS, Lyons T, Rock PB, Beidleman BA, et al (1995) Augmented Chemosensitivity At Altitude And After Return To Sea Level: Impact On Subsequent Return To Altitude. Acta Andina 4: 109–112.

[55] Alfrey CP, Rice L, Udden MM, Driscoll TB (1997) Neocytolysis: physiological down-regulator of red-cell mass. Lancet 349: 1389–1390. 9149714 10.1016/S0140-6736(96)09208-2

[56] Merino CF (1950) Studies on blood formation and destruction in the polycythemia of high altitude. Blood 5: 1–31. 15396777

[57] Rice L, Alfrey CP (2005) The negative regulation of red cell mass by neocytolysis: physiologic and pathophysiologic manifestations. Cellular Physiology and Biochemistry 15: 245–250. 16037689 10.1159/000087234

[58] Reynafarje C, Lozano R, Valdivieso J (1959) The polycythemia of high altitudes: iron metabolism and related aspects. Blood 14: 433–455. 13638344

[59] Robach P, Dechaux M, Jarrot S, Vaysse J, Schneider JC, et al (2000) Operation Everest III: role of plasma volume expansion on VO_2_max during prolonged high-altitude exposure. Journal of Applied Physiology 89: 29–37. 10904032 10.1152/jappl.2000.89.1.29

[60] Schneider M, Bernasch D, Weymann J, Holle R, Bartsch P (2002) Acute mountain sickness: influence of susceptibility, preexposure, and ascent rate. Medicine and Science in Sports and Exercise 34: 1886–1891. 12471292 10.1097/00005768-200212000-00005

[61] Richalet JP, Larmignat P, Poitrine E, Letournel M, Canoui-Poitrine F (2012) Physiological risk factors for severe high-altitude illness: a prospective cohort study. American Journal of Respiratory and Critical Care Medicine 185: 192–198. 22071330 10.1164/rccm.201108-1396OC

[62] Semenza GL (2011) Oxygen sensing, homeostasis, and disease. The New England Journal of Medicine 365: 537–547. 21830968 10.1056/NEJMra1011165

[63] Schuler B, Thomsen JJ, Gassmann M, Lundby C (2007) Timing the arrival at 2340 m altitude for aerobic performance. Scandinavian Journal of Medicine and Science in Sports 17: 588–594. 17316377 10.1111/j.1600-0838.2006.00611.x

[64] Lundby C, Damsgaard R (2006) Exercise performance in hypoxia after novel erythropoiesis stimulating protein treatment. Scandinavian Journal of Medicine and Science in Sports 16: 35–40. 16430679 10.1111/j.1600-0838.2004.00434.x

[65] Robach P, Calbet JA, Thomsen JJ, Boushel R, Mollard P, et al (2008) The ergogenic effect of recombinant human erythropoietin on VO_2_max depends on the severity of arterial hypoxemia. PLoS ONE 3: e2996. 18714372 10.1371/journal.pone.0002996 PMC2500186

[66] Young AJ, Sawka MN, Muza SR, Boushel R, Lyons T, et al (1996) Effects of erythrocyte infusion on VO_2_max at high altitude. Journal of Applied Physiology 81: 252–259. 8828672 10.1152/jappl.1996.81.1.252

[67] Janocha AJ, Koch CD, Tiso M, Ponchia A, Doctor A, et al (2011) Nitric oxide during altitude acclimatization. The New England Journal of Medicine 365: 1942–1944. 22087700 10.1056/NEJMc1107887 PMC3596889

[68] Nakhostine N, Lamontagne D (1993) Adenosine contributes to hypoxia-induced vasodilation through ATP-sensitive K+ channel activation. American Journal of Physiology 265: H1289–1293. 8238416 10.1152/ajpheart.1993.265.4.H1289

[69] Luft U, Opitz E (1942) Acclimatization studies on the Jungfraujoch: III. Increase in high altitude tolerance during and after acclimatization (translated from German). Luftfahrtmedizine 7: 205–217.

[70] Groves BM, Reeves JT, Sutton JR, Wagner PD, Cymerman A, et al (1987) Operation Everest II: elevated high-altitude pulmonary resistance unresponsive to oxygen. Journal of Applied Physiology 63: 521–530. 3654410 10.1152/jappl.1987.63.2.521

[71] Houston CS, Sutton JR, Cymerman A, Reeves JT (1987) Operation Everest II: man at extreme altitude. Journal of Applied Physiology 63: 877–882. 3654448 10.1152/jappl.1987.63.2.877

[72] Reeves JT, Groves BM, Sutton JR, Wagner PD, Cymerman A, et al (1987) Operation Everest II: preservation of cardiac function at extreme altitude. Journal of Applied Physiology 63: 531–539. 3654411 10.1152/jappl.1987.63.2.531

[73] Schoene RB, Roach RC, Hackett PH, Sutton JR, Cymerman A, et al (1990) Operation Everest II: ventilatory adaptation during gradual decompression to extreme altitude. Medicine and Science in Sports and Exercise 22: 804–810. 2287258

[74] Moore RJ, Friedl KE, Kramer TR, Martinez-Lopez LE, Hoyt RW (1992) Changes in soldier nutritional status and immune function during the Ranger training course. Defense Technical Information Center.

