# Supplementary material for: AltitudeOmics: The Integrative Physiology of Human Acclimatization to Hypobaric Hypoxia and Its Retention upon Reascent
Source: PLoS One. 2014 Mar 21;9(3):e92191. doi: 10.1371/journal.pone.0092191 (PMC3962396; doi:10.1371/journal.pone.0092191)
Supplement: Table S1 — Body Composition. Individual body weight data at SL, ALT1, ALT16, POST7 and POST21 and body fat and lean body mass at SL, ALT1, and ALT16. (PDF) [file pone.0092191.s001.pdf]

Table S1. Body Composition

ID	Body weight (kg)					Body fat (%)			Lean body mass (%)		
	SL	ALT1	ALT16	POST7	POST21	SL	ALT1	ALT16	SL	ALT1	ALT16
001	80.8		76.8		78.6	8.4	8.5	6.8	74.0		71.5
002	65.4		64.1		65.0	6.3	6.4	5.8	61.3		60.3
003	54.3		53.1		54.0	18.6	20.0	19.1	44.2		43.0
004	70.7		66.2		68.8	6.6	6.7	5.8	66.0		62.3
005	53.2	52.5	52.7		50.5	16.3	15.7	14.6	44.5	44.3	45.0
006	68.1	66.3	63.4		64.1	10.0	9.0	8.2	61.3	60.3	58.2
007	73.3	71.4	69.4		69.8	7.7	7.8	7.1	67.7	65.8	64.5
010	67.6	66.8	65.9	66.0		23.5	22.3	20.6	51.7	51.9	52.3
011	68.0	69.2	66.7	67.4		21.8	20.7	17.7	53.2	54.9	54.9
012	82.4	79.6	74.1	75.3		14.9	13.1	11.2	70.1	69.2	65.8
013	77.0	79.0	75.4	76.0		9.0	8.9	8.2	70.1	71.9	69.2
014	85.4	84.3	80.5	82.1		8.1	7.8	7.3	78.5	77.7	74.6
015	56.7	58.2	58.9	58.9		13.0	12.9	13.5	49.3	50.6	50.9
017	69.9	70.5	66.6	67.5		21.9	20.9	19.4	54.6	55.7	53.6
018	79.9	80.6	77.3	75.8		11.6	11.7	10.2	70.6	71.2	69.4
019	68.0	69.4	66.9	67.6		19.7	18.2	16.0	54.6	56.8	56.2
020	62.2	61.9	59.9	60.9		19.5	19.7	17.8	50.1	49.7	49.2
021	68.9	69.8	65.9	65.4		7.6	7.0	6.3	63.7	64.9	61.7
022	73.8	75.4	73.4	74.3		8.2	8.8	7.8	67.7	68.8	67.7
023	77.8	81.7	77.7	79.4		9.1	9.0	8.2	70.7	74.3	71.3
025	60.9	62.4	59.4	60.6		20.0	18.9	17.0	48.7	50.6	49.3

Table S2. Resting Arterial Blood Gases and Hemoglobin Concentration

ID	PaO_2 (mmHg)					$PaCO_2$ (mmHg)					SaO_2 (%)				
	SL	ALT1	ALT16	POST7	POST21	SL	ALT1	ALT16	POST7	POST21	SL	ALT1	ALT16	POST7	POST21
001	97.8	36.8	45.2		41.0	35.9	25.7	19.6		20.9	99	75	84		84
002	99.9					40.8					99				
003	109.3		45.7			33.4		18.8			99		82		
004	98.1		45.8		40.1	43.8		19.7		26.7	98		81		80
005	108.0	35.8	46.9		39.5	36.5	22.9	19.5		24.6	99	75	83		79
006	100.8	41.7	46.8		44.0	38.7	19.2	18.9		24.8	99	86	85		87
007	90.3	32.9	42.1		45.7	45.3	24.6	21.7		23.5	97	75	80		85
010	98.4	32.3	41.7	39.8		39.8	31.0	22.3	27.4		98	61	78	82	
011	103.4	35.8	46.7	44.0		38.7	26.3	20.6	24.7		98	75	83	81	
012	103.1	30.7	48.6	40.6		37.8	29.9	23.4	25.9		99	67	82	81	
013	102.3	34.9	44.4	42.5		39.6	26.4	19.2	23.5		98	76	82	82	
014	104.2	37.4	44.0	43.2		43.8	28.4	20.5	23.8		98	76	81	85	
015	113.9	36.2	41.4	46.6		*38.6	26.3	23.7	22.3		99	80	79	86	
017	109.8	38.3	55.4	52.7		33.4	24.9	13.8	17.2		99	88	91	92	
018	101.6	40.9	43.8	37.7		37.4	24.1	22.6	28.8		99	85	81	78	
019	98.9	33.8	44.4	41.6		42.9	29.5	22.4	24.4		98	72	79	80	
020	102.2	37.6	43.3	42.5		33.9	25.3	21.4	21.5		100	77	82	85	
021	96.5	36.1	48.8	39.0		38.9	26.6	19.1	25.4		98	76	86	77	
022	96.5	34.3	43.8	38.2		40.0	32.4	25.7	26.4		98	78	79	77	
023	104.9	35.1	44.7	38.2		38.9	28.1	23.1	27.8		99	78	83	80	
025	107.4	38.6	42.2	38.0		32.7	25.8	21.1	23.8		99	75	77	76	

ID	CaO_2 (ml/dL)					pH					P50 (mmHg)					Hemoglobin (g/dL)				
	SL	ALT1	ALT16	POST7	POST21	SL	ALT1	ALT16	POST7	POST21	SL	ALT1	ALT16	POST7	POST21	SL	ALT1	ALT16	POST7	POST21
001	20.6	15.6	19.4		17.4	7.39	7.51	7.49		7.54	18.8	27.8	27.4		25.8	14.8	14.9	16.6		14.8
002	20.1					7.42					20.9					14.5				
003	18.3		16.8			7.42		7.49			18.0		29.1			13.1		14.7		
004	21.1		21.4		18.3	7.38		7.49		7.48	22.8		29.8		26.3	15.3		18.9		16.3
005	18.0	13.6	14.8		13.9	7.38	7.52	7.52		7.49	20.5	27.4	30.0		27.1	12.9	13.0	12.7		12.6
006	21.0	18.8	21.3		19.7	7.39	7.53	7.51		7.50	19.7	24.7	27.9		24.3	15.1	15.6	17.9		16.2
007	20.6	16.7	21.0		18.7	7.37	7.55	7.51		7.54	25.0	26.0	28.9		28.4	15.2	16.0	18.8		15.8
010	16.4	10.2	14.6	13.2		7.41	7.48	7.50	7.47		25.4	30.0	29.6	24.7		11.9	11.9	13.4	11.5	
011	17.5	13.1	16.3	14.1		7.41	7.50	7.50	7.49		24.3	26.9	29.0	28.5		12.6	12.5	14.0	12.4	
012	19.1	14.4	20.0	17.2		7.36	7.48	7.48	7.50		19.8	25.8	30.1	26.6		13.7	15.4	17.4	15.2	
013	22.1	15.9	20.0	18.3		7.41	7.52	7.50	7.52		23.0	26.1	28.3	28.1		16.0	14.9	17.5	16.0	
014	21.8	17.7	20.6	19.3		7.40	7.50	7.52	7.54		24.1	27.1	29.6	26.3		15.8	16.6	18.2	16.2	
015	16.7	14.3	15.1	16.2		7.49	7.54	7.50	7.51		17.9	25.6	28.5	26.7		11.8	12.9	13.7	13.4	
017	17.7	15.0	18.7	16.8		7.40	7.49	7.52	7.55		20.7	20.1	27.2	25.4		12.6	12.2	14.7	13.0	
018	19.7	17.3	19.0	16.7		7.39	7.51	7.50	7.51		21.3	24.5	28.6	27.0		14.2	14.6	16.8	15.4	
019	16.1	12.1	14.3	12.6		7.40	7.47	7.47	7.49		22.5	25.8	29.2	27.4		11.6	12.0	12.8	11.2	
020	18.1	15.1	17.9	16.1		7.43	7.49	7.50	7.50		13.2	26.9	27.7	25.2		12.9	14.0	15.7	13.6	
021	21.3	16.3	19.7	16.2		7.43	7.51	7.49	7.50		23.6	26.5	27.3	28.2		15.4	15.3	16.3	15.2	
022	21.2	16.3	20.2	16.7		7.41	7.45	7.47	7.49		22.9	23.0	28.7	26.8		15.3	15.1	18.2	15.4	
023	20.4	16.4	20.5	17.4		7.40	7.49	7.46	7.50		18.6	24.7	26.8	25.6		14.6	15.1	17.8	15.6	
025	18.6	14.6	16.4	13.8		7.44	7.55	7.51	7.51		23.4	30.4	30.4	28.2		13.3	13.8	15.2	13.0	

Table S3. Acute Mountain Sickness Scores for Lake Louise (LLQ) and Environmental Symptom (AMS-C) Questionnaires

ID	LLQ - Fatigue					LLQ - Headache					LLQ - Gastrointestinal					LLQ - Dizzy					LLQ AMS Score					AMS-C - Composite Score				
	SL	ALT1	ALT16	POST7	POST21	SL	ALT1	ALT16	POST7	POST21	SL	ALT1	ALT16	POST7	POST21	SL	ALT1	ALT16	POST7	POST21	SL	ALT1	ALT16	POST7	POST21	SL	ALT1	ALT16	POST7	POST21
001	0	2	0		2	0	2	0		1	0	1	0		0	0	2	0		1	0	7	0		4	0.0	3.5	0.1		1.4
002	0	1	0		1	0	1	0		1	0	0	0		0	0	0	0		0	0	2	0		2	0.0	0.4	0.0		0.3
003	0	0	2		1	0	1	0		1	0	0	0		1	1	1	1		1	0	2	0		4	0.0	0.0	0.1		1.1
004	1	2	1		2	0	3	0		0	1	2	0		0	1	1	1		1	0	8	0		0	0.0	3.3	0.0		0.4
005	1	2	1		1	0	1	0		0	0	0	0		0	0	1	1		0	0	4	0		0	0.0	0.9	0.2		0.1
006	0	3	2		1	0	2	0		1	0	2	0		1	0	1	1		1	0	8	0		4	0.0	2.3	0.3		0.6
007	0	2	0		1	0	2	0		1	0	0	0		0	0	1	0		0	0	5	0		2	0.0	2.6	0.0		0.3
010	0	2	1	1		0	2	0	0		0	0	0	0		0	2	0	1		0	6	0	0		0.0	1.7	0.2	0.3	
011	0	2	1	1		0	2	0	0		0	1	1	0		0	1	0	0		0	6	0	0		0.0	1.1	0.4	0.1	
012	0	1	1	0		0	0	0	0		0	0	0	0		0	0	0	0		0	0	0	0		0.1	0.0	0.3	0.0	
013	0	2	1	0		0	2	0	0		0	3	2	0		0	1	0	0		0	8	0	0		0.0	2.6	0.5	0.1	
014	0	1	0	0		0	2	0	0		0	0	0	0		0	0	0	0		0	3	0	0		0.0	1.2	0.2	0.3	
015	0	3	1	1		0	3	0	0		0	3	0	2		0	2	0	2		0	11	0	0		0.0	3.0	0.1	0.5	
017	1	3	1	1		0	3	0	0		0	2	0	0		0	2	1	0		0	10	0	0		0.2	3.8	0.8	0.0	
018	0	2	1	1		0	2	0	0		0	0	0	0		0	1	0	0		0	5	0	0		0.0	1.0	0.0	0.0	
019	1	2	1	1		0	2	0	0		0	1	0	0		0	1	1	0		0	6	0	0		0.3	1.2	0.1	0.0	
020	0	2	2	1		0	2	0	0		0	0	0	1		0	1	0	1		0	5	0	0		0.0	2.8	0.4	0.6	
021	0	0	0	0		0	0	0	0		0	1	0	0		0	0	0	0		0	0	0	0		0.0	0.5	0.0	0.0	
022	0	3	1	0		0	3	0	0		0	1	0	0		0	1	0	0		0	8	0	0		0.0	1.8	0.1	0.1	
023	0	1	0	1		0	2	0	0		0	0	0	0		0	1	0	0		0	4	0	0		0.0	0.9	0.0	0.2	
025	1	2	1	2		0	2	0	0		0	1	0	0		0		1	0		0	5	0	0		0.2	2.4	0.1	0.0	

Table S4a. Cognitive Function Tests

ID	SRT-1					SRT-2					CodeSub-CDS					CodeSub-CSD				
	SL	ALT1	ALT16	POST7	POST21	SL	ALT1	ALT16	POST7	POST21	SL	ALT1	ALT16	POST7	POST21	SL	ALT1	ALT16	POST7	POST21
001	196.77	163.93	154.43		144.39	186.95	108.71	145.33		130.24	41.54	44.18	42.72		45.36	0.78	0.89	0.92		0.94
002	232.18	179.35	202.74		176.86	189.49	169.83	167.33		162.51	47.85	38.87	43.92		40.58	1.00	1.00	1.00		1.00
003	185.82	146.64	128.52		155.78	188.44	116.42	162.93		157.16	54.18	47.69	53.61		56.08	0.96	0.94	1.00		0.97
004	180.14	169.79	195.09		178.77	178.52	162.60	169.59		177.02	52.49	49.58	61.68		53.83	0.96	0.94	0.96		0.94
005	217.26	197.38	189.72		182.22	200.45	182.26	190.46		161.20	49.27	50.40	47.52		46.29	0.93	0.99	0.88		0.93
006	212.69	215.32	187.51		194.50	212.61	180.38	188.87		200.22	62.19	50.84	57.07		58.57	0.98	0.85	0.94		0.97
007	217.43	195.65	231.35		218.20	220.64	216.72	213.03		216.08	46.66	38.72	46.28		49.37	0.95	0.97	0.96		0.90
010	197.79	167.14	173.72	170.03		178.43	169.58	171.00	129.61		56.83	58.06	62.17	55.67		0.97	0.99	0.99	0.93	
011	198.13	175.19	185.77	183.87		208.09	145.71	185.25	188.58		48.11	43.77	50.46	48.90		0.92	0.85	0.96	0.99	
012	170.19	134.87	164.09	141.68		167.81	85.62	164.29	143.93		49.04	40.08	49.07	46.20		0.99	0.99	0.94	1.00	
013	224.13	229.07	211.40	194.30		217.31	188.06	209.39	144.88		51.10	49.49	50.98	52.22		0.93	0.88	0.99	0.96	
014	235.03	227.45	237.67	240.38		236.56	203.79	227.77	166.12		63.11	63.44	59.26	61.65		0.97	0.99	0.99	0.97	
015	178.46	32.26	135.27	161.21		175.57	96.02	164.39	140.15		44.95	31.52	46.12	43.79		0.85	0.61	0.92	0.89	
017	194.65	164.79	202.02	159.76		171.19	132.75	178.29	132.62		57.31	41.54	57.14	48.99		0.83	0.90	0.92	0.90	
018	219.10	166.54	246.10	209.52		211.93	173.62	236.29	200.65		47.76	42.68	59.96	46.01		0.96	0.65	1.00	0.86	
019	214.63	171.81	205.30	191.10		205.81	135.71	185.90	175.37		62.85	53.08	62.27	54.64		0.90	0.82	1.00	0.92	
020	156.90	77.32	136.60	134.26		158.02	103.53	123.61	150.90		41.62	38.43	41.92	47.61		0.82	0.76	0.89	0.96	
021	189.10	199.92	222.55	162.73		201.33	139.08	206.24	145.78		45.16	37.80	47.91	42.38		0.99	0.93	0.99	0.93	
022	178.42	203.17	207.61	191.54		180.72	181.27	200.23	173.64		53.43	64.64	66.91	58.30		0.97	0.82	1.00	0.96	
023	222.60	195.30	196.53	208.82		202.70	151.07	199.00	121.41		51.21	53.71	55.52	58.88		0.91	0.81	0.93	0.99	
025	223.48	165.16	196.67	190.73		197.29	159.08	184.02	166.27		55.94	57.30	58.05	61.53		0.94	0.94	0.97	0.94	

Table S4b. Cognitive Function Tests

ID	Spatial					Go/NoGo					Memory Search					Match2Sample				
	**SL**	**ALT1**	**ALT16**	**POST7**	**POST21**	**SL**	**ALT1**	**ALT16**	**POST7**	**POST21**	**SL**	**ALT1**	**ALT16**	**POST7**	**POST21**	**SL**	**ALT1**	**ALT16**	**POST7**	**POST21**
001	40.82	26.13	27.52		39.70	120.18	113.95	105.31		118.58	70.82	22.46	68.52		57.41	31.55	6.23	34.95		35.04
002	46.44	36.20	38.91		43.65	145.51	140.32	133.41		130.21	53.40	50.78	50.23		35.47	45.98	33.48	34.91		29.13
003	36.17	27.03	32.42		39.33	124.17	104.29	129.63		115.45	77.71	68.43	70.10		82.17	34.27	21.32	45.59		31.15
004	48.97	31.51	59.33		37.97	115.89	113.78	136.01		110.61	85.90	44.80	90.60		85.48	36.41	22.38	23.49		28.76
005	37.90	38.09	36.45		43.06	140.12	133.45	131.41		120.92	72.20	65.21	73.78		76.80	31.53	22.19	34.54		34.21
006	46.75	53.22	53.22		27.69	130.06	140.19	128.45		110.64	74.38	66.39	78.28		83.46	38.85	35.98	41.08		43.21
007	41.65	36.73	48.72		51.24	127.67	112.94	151.36		136.12	72.87	78.36	89.65		65.29	38.08	34.05	46.37		33.99
010	32.12	34.21	30.86	30.14		116.46	102.88	97.39	95.34		86.06	104.19	74.72	81.71		35.23	23.74	26.69	32.79	
011	36.23	37.39	36.25	35.98		130.61	122.97	128.96	126.44		62.56	77.49	59.03	63.31		47.71	38.23	34.94	38.58	
012	37.75	41.07	48.44	38.21		112.29	113.31	108.36	114.27		59.29	46.03	70.05	71.02		38.75	25.57	37.59	34.36	
013	39.24	37.41	38.16	48.79		137.36	145.77	136.16	122.27		86.13	65.85	76.48	77.07		33.36	31.54	26.84	26.70	
014	50.33	47.82	54.44	41.05		142.27	156.88	144.62	146.91		93.34	89.74	89.98	85.77		40.92	34.04	43.49	30.77	
015	41.02	34.77	42.74	54.02		113.63	85.64	118.66	110.40		75.11	37.48	76.11	70.60		32.38	12.85	36.25	9.66	
017	36.27	34.97	42.74	37.78		118.58	123.00	137.33	120.40		87.42	79.99	94.64	50.78		38.59	33.13	34.26	43.25	
018	47.52	33.28	45.32	34.80		119.47	91.97	142.07	137.05		71.71	8.52	90.15	25.55		24.54	3.50	28.96	36.56	
019	38.72	42.01	59.05	46.36		147.59	147.06	144.33	135.91		70.77	87.19	95.08	58.42		37.26	42.08	46.38	42.26	
020	38.07	35.08	38.25	36.73		99.49	83.58	106.26	114.80		50.17	54.59	50.22	41.52		21.81	24.38	29.19	28.50	
021	40.50	37.46	51.52	36.04		96.47	105.50	132.62	90.68		81.26	59.41	85.98	61.66		27.56	23.81	30.47	24.85	
022	47.88	39.09	57.19	52.12		105.19	127.95	122.55	117.55		63.70	70.10	68.40	57.41		30.50	27.45	31.27	26.30	
023	35.64	34.56	37.93	33.09		119.68	115.89	140.30	103.25		54.15	52.22	71.70	57.27		27.83	24.88	32.46	21.60	
025	46.08	53.24	56.76	30.90		136.62	123.07	145.37	130.25		67.15	84.79	90.20	84.26		72.88	52.74	71.79	63.28	

Table S4c. Cognitive Function Tests

ID	ProcRT				
	SL	ALT1	ALT16	POST7	POST21
001	92.85	81.93	94.56		109.61
002	105.70	97.14	115.15		93.88
003	113.04	97.81	101.30		105.67
004	106.51	80.87	113.54		103.99
005	135.20	110.15	111.76		118.33
006	107.82	106.14	111.56		103.97
007	117.44	112.17	121.90		123.52
010	103.20	105.25	108.14	109.30	
011	114.48	81.98	108.11	104.38	
012	93.79	92.70	103.09	90.73	
013	108.27	102.15	109.59	110.38	
014	133.72	128.88	128.63	113.06	
015	98.66	84.09	99.94	88.36	
017	115.39	96.69	114.82	94.95	
018	101.44	70.19	120.57	103.15	
019	120.54	108.55	115.19	116.33	
020	86.75	78.94	93.82	89.73	
021	111.52	95.01	110.52	70.73	
022	101.63	114.59	118.98	109.46	
023	110.94	100.51	108.89	108.24	
025	121.17	124.26	120.71	110.78	

Table S5. Peak Power Output and Submaximal Exercise Performance

ID	VO_{2max} (l/min)			Peak Power Output (W)			5km Time to Completion (s)			Running speed (m/s)				
	SL	ALT1	ALT16	SL	ALT1	ALT16	SL	ALT1	ALT16	SL	ALT1	ALT16	POST7	POST21
001	3.52	2.60	2.95	310	190	227	480	800	601	3.19	1.46	1.78		1.74
002	4.07		2.95	353		250	484		587	3.79	2.18	2.16		2.21
003	2.04		1.59	160		100	677		881	2.21	1.43	1.40		
004	4.28		2.80	327		190	484		613	3.83	2.00	2.14		2.03
005	2.24	1.56	1.87	181	130	130	636	853	789	2.35	1.45	1.45		1.39
006	3.57		2.70	265		160	566		746	2.92	1.79	1.98		1.82
007	5.00		3.23	330		250	489		655	3.55	1.94	2.22		2.14
010	2.59	2.00	1.88	225	160	160	700	805	791	2.89	1.43	1.44	1.44	
011	3.46	2.13	2.47	239	160	160	558	653	659	2.78	1.66	1.70	1.76	
012	3.52	2.49	2.52	268	160	160	557	641	719	2.51	1.53	1.49	1.76	
013	3.64	2.92	3.07	285	212	212	558	642	706	2.68	1.67	1.84	1.80	
014	4.75	3.26	2.72	330	235	220	531	646	601	2.80	1.54	1.79	1.80	
015	3.14	1.35	1.46	213	100	100	586	856	807	2.86	1.49	1.81	1.63	
017	3.19	2.01	2.13	232	145	145	568	706	988	2.29	1.20	1.36	1.37	
018	3.40	2.59	2.99	257	167	160	539	627	684	2.70	1.55	1.72	1.54	
019	2.72	1.74	1.87	220	130	145	581	733	800	1.91		1.16	1.13	
020	2.18	1.93	2.03	220	160	160	715	763	748	2.77	1.47	1.46	1.34	
021	4.27		2.99	300	205	198	497	637	573	3.16			1.91	
022	4.10	3.05	2.66	302	212	220	531	650	655	3.31	1.65	2.00	1.95	
023	3.75	3.06	2.93	355	235	235		599	618	2.86	1.78	1.90	1.79	
025	2.38	1.89	2.16	198	130	130	643	733	789	2.29	1.36	1.42	1.55	
