# Supplementary material for: AltitudeOmics: The Integrative Physiology of Human Acclimatization to Hypobaric Hypoxia and Its Retention upon Reascent
Source: PLoS One. 2014 Mar 21;9(3):e92191. doi: 10.1371/journal.pone.0092191 (PMC3962396; doi:10.1371/journal.pone.0092191)
Supplement: Table S2 — Resting Arterial Blood Gases and Hemoglobin Concentration. Individual resting arterial blood gases and [Hb] data at SL, ALT1, ALT16, POST7 and POST21. (PDF) [file pone.0092191.s002.pdf]

Table S2. Resting Arterial Blood Gases and Hemoglobin Concentration

ID	PaO_2 (mmHg)					$PaCO_2$ (mmHg)					SaO_2 (%)					CaO_2 (ml/dL)					pH					P50 (mmHg)					Hemoglobin (g/dL)				
	SL	ALT1	ALT16	POST7	POST21	SL	ALT1	ALT16	POST7	POST21	SL	ALT1	ALT16	POST7	POST21	SL	ALT1	ALT16	POST7	POST21	SL	ALT1	ALT16	POST7	POST21	SL	ALT1	ALT16	POST7	POST21	SL	ALT1	ALT16	POST7	POST21
001	97.8	36.8	45.2		41.0	35.9	25.7	19.6		20.9	99	75	84		84	20.6	15.6	19.4		17.4	7.39	7.51	7.49		7.54	18.8	27.8	27.4		25.8	14.8	14.9	16.6		14.8
002	99.9					40.8					99					20.1					7.42					20.9					14.5				
003	109.3		45.7			33.4		18.8			99		82			18.3		16.8			7.42		7.49			18.0		29.1			13.1		14.7		
004	98.1		45.8		40.1	43.8		19.7		26.7	98		81		80	21.1		21.4		18.3	7.38		7.49		7.48	22.8		29.8		26.3	15.3		18.9		16.3
005	108.0	35.8	46.9		39.5	36.5	22.9	19.5		24.6	99	75	83		79	18.0	13.6	14.8		13.9	7.38	7.52	7.52		7.49	20.5	27.4	30.0		27.1	12.9	13.0	12.7		12.6
006	100.8	41.7	46.8		44.0	38.7	19.2	18.9		24.8	99	86	85		87	21.0	18.8	21.3		19.7	7.39	7.53	7.51		7.50	19.7	24.7	27.9		24.3	15.1	15.6	17.9		16.2
007	90.3	32.9	42.1		45.7	45.3	24.6	21.7		23.5	97	75	80		85	20.6	16.7	21.0		18.7	7.37	7.55	7.51		7.54	25.0	26.0	28.9		28.4	15.2	16.0	18.8		15.8
010	98.4	32.3	41.7	39.8		39.8	31.0	22.3	27.4		98	61	78	82		16.4	10.2	14.6	13.2		7.41	7.48	7.50	7.47		25.4	30.0	29.6	24.7		11.9	11.9	13.4	11.5	
011	103.4	35.8	46.7	44.0		38.7	26.3	20.6	24.7		98	75	83	81		17.5	13.1	16.3	14.1		7.41	7.50	7.50	7.49		24.3	26.9	29.0	28.5		12.6	12.5	14.0	12.4	
012	103.1	30.7	48.6	40.6		37.8	29.9	23.4	25.9		99	67	82	81		19.1	14.4	20.0	17.2		7.36	7.48	7.48	7.50		19.8	25.8	30.1	26.6		13.7	15.4	17.4	15.2	
013	102.3	34.9	44.4	42.5		39.6	26.4	19.2	23.5		98	76	82	82		22.1	15.9	20.0	18.3		7.41	7.52	7.50	7.52		23.0	26.1	28.3	28.1		16.0	14.9	17.5	16.0	
014	104.2	37.4	44.0	43.2		43.8	28.4	20.5	23.8		98	76	81	85		21.8	17.7	20.6	19.3		7.40	7.50	7.52	7.54		24.1	27.1	29.6	26.3		15.8	16.6	18.2	16.2	
015	113.9	36.2	41.4	46.6		*38.6	26.3	23.7	22.3		99	80	79	86		16.7	14.3	15.1	16.2		7.49	7.54	7.50	7.51		17.9	25.6	28.5	26.7		11.8	12.9	13.7	13.4	
017	109.8	38.3	55.4	52.7		33.4	24.9	13.8	17.2		99	88	91	92		17.7	15.0	18.7	16.8		7.40	7.49	7.52	7.55		20.7	20.1	27.2	25.4		12.6	12.2	14.7	13.0	
018	101.6	40.9	43.8	37.7		37.4	24.1	22.6	28.8		99	85	81	78		19.7	17.3	19.0	16.7		7.39	7.51	7.50	7.51		21.3	24.5	28.6	27.0		14.2	14.6	16.8	15.4	
019	98.9	33.8	44.4	41.6		42.9	29.5	22.4	24.4		98	72	79	80		16.1	12.1	14.3	12.6		7.40	7.47	7.47	7.49		22.5	25.8	29.2	27.4		11.6	12.0	12.8	11.2	
020	102.2	37.6	43.3	42.5		33.9	25.3	21.4	21.5		100	77	82	85		18.1	15.1	17.9	16.1		7.43	7.49	7.50	7.50		13.2	26.9	27.7	25.2		12.9	14.0	15.7	13.6	
021	96.5	36.1	48.8	39.0		38.9	26.6	19.1	25.4		98	76	86	77		21.3	16.3	19.7	16.2		7.43	7.51	7.49	7.50		23.6	26.5	27.3	28.2		15.4	15.3	16.3	15.2	
022	96.5	34.3	43.8	38.2		40.0	32.4	25.7	26.4		98	78	79	77		21.2	16.3	20.2	16.7		7.41	7.45	7.47	7.49		22.9	23.0	28.7	26.8		15.3	15.1	18.2	15.4	
023	104.9	35.1	44.7	38.2		38.9	28.1	23.1	27.8		99	78	83	80		20.4	16.4	20.5	17.4		7.40	7.49	7.46	7.50		18.6	24.7	26.8	25.6		14.6	15.1	17.8	15.6	
025	107.4	38.6	42.2	38.0		32.7	25.8	21.1	23.8		99	75	77	76		18.6	14.6	16.4	13.8		7.44	7.55	7.51	7.51		23.4	30.4	30.4	28.2		13.3	13.8	15.2	13.0	
