# Supplementary material for: AltitudeOmics: The Integrative Physiology of Human Acclimatization to Hypobaric Hypoxia and Its Retention upon Reascent
Source: PLoS One. 2014 Mar 21;9(3):e92191. doi: 10.1371/journal.pone.0092191 (PMC3962396; doi:10.1371/journal.pone.0092191)
Supplement: Table S3 — Acute Mountain Sickness Scores for Lake Louise (LLQ) and Environmental Symptom (AMS-C) Questionnaires. Individual AMS symptom scores and the composite LL and AMS-C scores at SL, ALT1, ALT16, POST7 and POST21. (PDF) [file pone.0092191.s003.pdf]

Table S3. Acute Mountain Sickness Scores for Lake Louise (LLQ) and Environmental Symptom (AMS-C) Questionnaires

ID	LLQ - Fatigue					LLQ - Headache					LLQ - Gastrointestinal					LLQ - Dizzy					LLQ AMS Score					AMS-C - Composite Score				
	SL	ALT1	ALT16	POST7	POST21	SL	ALT1	ALT16	POST7	POST21	SL	ALT1	ALT16	POST7	POST21	SL	ALT1	ALT16	POST7	POST21	SL	ALT1	ALT16	POST7	POST21	SL	ALT1	ALT16	POST7	POST21
001	0	2	0		2	0	2	0		1	0	1	0		0	0	2	0		1	0	7	0		4	0.0	3.5	0.1		1.4
002	0	1	0		1	0	1	0		1	0	0	0		0	0	0	0		0	0	2	0		2	0.0	0.4	0.0		0.3
003	0	0	2		1	0	1	0		1	0	0	0		1	1	1	1		1	0	2	0		4	0.0	0.0	0.1		1.1
004	1	2	1		2	0	3	0		0	1	2	0		0	1	1	1		1	0	8	0		0	0.0	3.3	0.0		0.4
005	1	2	1		1	0	1	0		0	0	0	0		0	0	1	1		0	0	4	0		0	0.0	0.9	0.2		0.1
006	0	3	2		1	0	2	0		1	0	2	0		1	0	1	1		1	0	8	0		4	0.0	2.3	0.3		0.6
007	0	2	0		1	0	2	0		1	0	0	0		0	0	1	0		0	0	5	0		2	0.0	2.6	0.0		0.3
010	0	2	1	1		0	2	0	0		0	0	0	0		0	2	0	1		0	6	0	0		0.0	1.7	0.2	0.3	
011	0	2	1	1		0	2	0	0		0	1	1	0		0	1	0	0		0	6	0	0		0.0	1.1	0.4	0.1	
012	0	1	1	0		0	0	0	0		0	0	0	0		0	0	0	0		0	0	0	0		0.1	0.0	0.3	0.0	
013	0	2	1	0		0	2	0	0		0	3	2	0		0	1	0	0		0	8	0	0		0.0	2.6	0.5	0.1	
014	0	1	0	0		0	2	0	0		0	0	0	0		0	0	0	0		0	3	0	0		0.0	1.2	0.2	0.3	
015	0	3	1	1		0	3	0	0		0	3	0	2		0	2	0	2		0	11	0	0		0.0	3.0	0.1	0.5	
017	1	3	1	1		0	3	0	0		0	2	0	0		0	2	1	0		0	10	0	0		0.2	3.8	0.8	0.0	
018	0	2	1	1		0	2	0	0		0	0	0	0		0	1	0	0		0	5	0	0		0.0	1.0	0.0	0.0	
019	1	2	1	1		0	2	0	0		0	1	0	0		0	1	1	0		0	6	0	0		0.3	1.2	0.1	0.0	
020	0	2	2	1		0	2	0	0		0	0	0	1		0	1	0	1		0	5	0	0		0.0	2.8	0.4	0.6	
021	0	0	0	0		0	0	0	0		0	1	0	0		0	0	0	0		0	0	0	0		0.0	0.5	0.0	0.0	
022	0	3	1	0		0	3	0	0		0	1	0	0		0	1	0	0		0	8	0	0		0.0	1.8	0.1	0.1	
023	0	1	0	1		0	2	0	0		0	0	0	0		0	1	0	0		0	4	0	0		0.0	0.9	0.0	0.2	
025	1	2	1	2		0	2	0	0		0	1	0	0		0		1	0		0	5	0	0		0.2	2.4	0.1	0.0	
