# Supplementary material for: AltitudeOmics: The Integrative Physiology of Human Acclimatization to Hypobaric Hypoxia and Its Retention upon Reascent
Source: PLoS One. 2014 Mar 21;9(3):e92191. doi: 10.1371/journal.pone.0092191 (PMC3962396; doi:10.1371/journal.pone.0092191)
Supplement: Table S4 — a. Cognitive Function Tests. Individual cognitive function test scores for Simple Reaction Time-1, Simple Reaction Time-2, Code Substitution—Simultaneous, and Code Substitution—Delayed Recall at SL, ALT1, ALT16, POST7 and POST21. b. Cognitive Function Tests. Individual cognitive function test scores for Spatial Discrimination, Go-No-Go, Sternberg's Memory Search, and Matching to Sample at SL, ALT1, ALT16, POST7 and POST21. c. Cognitive Function Tests. Individual cognitive function test score for Procedural Reaction Time at SL, ALT1, ALT16, POST7 and POST21. (PDF) [file pone.0092191.s004.pdf]

Table S4a. Cognitive Function Tests

ID	SRT-1					SRT-2					CodeSub-CDS					CodeSub-CSD				
	SL	ALT1	ALT16	POST7	POST21	SL	ALT1	ALT16	POST7	POST21	SL	ALT1	ALT16	POST7	POST21	SL	ALT1	ALT16	POST7	POST21
001	196.77	163.93	154.43		144.39	186.95	108.71	145.33		130.24	41.54	44.18	42.72		45.36	0.78	0.89	0.92		0.94
002	232.18	179.35	202.74		176.86	189.49	169.83	167.33		162.51	47.85	38.87	43.92		40.58	1.00	1.00	1.00		1.00
003	185.82	146.64	128.52		155.78	188.44	116.42	162.93		157.16	54.18	47.69	53.61		56.08	0.96	0.94	1.00		0.97
004	180.14	169.79	195.09		178.77	178.52	162.60	169.59		177.02	52.49	49.58	61.68		53.83	0.96	0.94	0.96		0.94
005	217.26	197.38	189.72		182.22	200.45	182.26	190.46		161.20	49.27	50.40	47.52		46.29	0.93	0.99	0.88		0.93
006	212.69	215.32	187.51		194.50	212.61	180.38	188.87		200.22	62.19	50.84	57.07		58.57	0.98	0.85	0.94		0.97
007	217.43	195.65	231.35		218.20	220.64	216.72	213.03		216.08	46.66	38.72	46.28		49.37	0.95	0.97	0.96		0.90
010	197.79	167.14	173.72	170.03		178.43	169.58	171.00	129.61		56.83	58.06	62.17	55.67		0.97	0.99	0.99	0.93	
011	198.13	175.19	185.77	183.87		208.09	145.71	185.25	188.58		48.11	43.77	50.46	48.90		0.92	0.85	0.96	0.99	
012	170.19	134.87	164.09	141.68		167.81	85.62	164.29	143.93		49.04	40.08	49.07	46.20		0.99	0.99	0.94	1.00	
013	224.13	229.07	211.40	194.30		217.31	188.06	209.39	144.88		51.10	49.49	50.98	52.22		0.93	0.88	0.99	0.96	
014	235.03	227.45	237.67	240.38		236.56	203.79	227.77	166.12		63.11	63.44	59.26	61.65		0.97	0.99	0.99	0.97	
015	178.46	32.26	135.27	161.21		175.57	96.02	164.39	140.15		44.95	31.52	46.12	43.79		0.85	0.61	0.92	0.89	
017	194.65	164.79	202.02	159.76		171.19	132.75	178.29	132.62		57.31	41.54	57.14	48.99		0.83	0.90	0.92	0.90	
018	219.10	166.54	246.10	209.52		211.93	173.62	236.29	200.65		47.76	42.68	59.96	46.01		0.96	0.65	1.00	0.86	
019	214.63	171.81	205.30	191.10		205.81	135.71	185.90	175.37		62.85	53.08	62.27	54.64		0.90	0.82	1.00	0.92	
020	156.90	77.32	136.60	134.26		158.02	103.53	123.61	150.90		41.62	38.43	41.92	47.61		0.82	0.76	0.89	0.96	
021	189.10	199.92	222.55	162.73		201.33	139.08	206.24	145.78		45.16	37.80	47.91	42.38		0.99	0.93	0.99	0.93	
022	178.42	203.17	207.61	191.54		180.72	181.27	200.23	173.64		53.43	64.64	66.91	58.30		0.97	0.82	1.00	0.96	
023	222.60	195.30	196.53	208.82		202.70	151.07	199.00	121.41		51.21	53.71	55.52	58.88		0.91	0.81	0.93	0.99	
025	223.48	165.16	196.67	190.73		197.29	159.08	184.02	166.27		55.94	57.30	58.05	61.53		0.94	0.94	0.97	0.94	

Table S4b. Cognitive Function Tests

ID	Spatial					Go/NoGo					Memory Search					Match2Sample				
	**SL**	**ALT1**	**ALT16**	**POST7**	**POST21**	**SL**	**ALT1**	**ALT16**	**POST7**	**POST21**	**SL**	**ALT1**	**ALT16**	**POST7**	**POST21**	**SL**	**ALT1**	**ALT16**	**POST7**	**POST21**
001	40.82	26.13	27.52		39.70	120.18	113.95	105.31		118.58	70.82	22.46	68.52		57.41	31.55	6.23	34.95		35.04
002	46.44	36.20	38.91		43.65	145.51	140.32	133.41		130.21	53.40	50.78	50.23		35.47	45.98	33.48	34.91		29.13
003	36.17	27.03	32.42		39.33	124.17	104.29	129.63		115.45	77.71	68.43	70.10		82.17	34.27	21.32	45.59		31.15
004	48.97	31.51	59.33		37.97	115.89	113.78	136.01		110.61	85.90	44.80	90.60		85.48	36.41	22.38	23.49		28.76
005	37.90	38.09	36.45		43.06	140.12	133.45	131.41		120.92	72.20	65.21	73.78		76.80	31.53	22.19	34.54		34.21
006	46.75	53.22	53.22		27.69	130.06	140.19	128.45		110.64	74.38	66.39	78.28		83.46	38.85	35.98	41.08		43.21
007	41.65	36.73	48.72		51.24	127.67	112.94	151.36		136.12	72.87	78.36	89.65		65.29	38.08	34.05	46.37		33.99
010	32.12	34.21	30.86	30.14		116.46	102.88	97.39	95.34		86.06	104.19	74.72	81.71		35.23	23.74	26.69	32.79	
011	36.23	37.39	36.25	35.98		130.61	122.97	128.96	126.44		62.56	77.49	59.03	63.31		47.71	38.23	34.94	38.58	
012	37.75	41.07	48.44	38.21		112.29	113.31	108.36	114.27		59.29	46.03	70.05	71.02		38.75	25.57	37.59	34.36	
013	39.24	37.41	38.16	48.79		137.36	145.77	136.16	122.27		86.13	65.85	76.48	77.07		33.36	31.54	26.84	26.70	
014	50.33	47.82	54.44	41.05		142.27	156.88	144.62	146.91		93.34	89.74	89.98	85.77		40.92	34.04	43.49	30.77	
015	41.02	34.77	42.74	54.02		113.63	85.64	118.66	110.40		75.11	37.48	76.11	70.60		32.38	12.85	36.25	9.66	
017	36.27	34.97	42.74	37.78		118.58	123.00	137.33	120.40		87.42	79.99	94.64	50.78		38.59	33.13	34.26	43.25	
018	47.52	33.28	45.32	34.80		119.47	91.97	142.07	137.05		71.71	8.52	90.15	25.55		24.54	3.50	28.96	36.56	
019	38.72	42.01	59.05	46.36		147.59	147.06	144.33	135.91		70.77	87.19	95.08	58.42		37.26	42.08	46.38	42.26	
020	38.07	35.08	38.25	36.73		99.49	83.58	106.26	114.80		50.17	54.59	50.22	41.52		21.81	24.38	29.19	28.50	
021	40.50	37.46	51.52	36.04		96.47	105.50	132.62	90.68		81.26	59.41	85.98	61.66		27.56	23.81	30.47	24.85	
022	47.88	39.09	57.19	52.12		105.19	127.95	122.55	117.55		63.70	70.10	68.40	57.41		30.50	27.45	31.27	26.30	
023	35.64	34.56	37.93	33.09		119.68	115.89	140.30	103.25		54.15	52.22	71.70	57.27		27.83	24.88	32.46	21.60	
025	46.08	53.24	56.76	30.90		136.62	123.07	145.37	130.25		67.15	84.79	90.20	84.26		72.88	52.74	71.79	63.28	

Table S4c. Cognitive Function Tests

ID	ProcRT				
	SL	ALT1	ALT16	POST7	POST21
001	92.85	81.93	94.56		109.61
002	105.70	97.14	115.15		93.88
003	113.04	97.81	101.30		105.67
004	106.51	80.87	113.54		103.99
005	135.20	110.15	111.76		118.33
006	107.82	106.14	111.56		103.97
007	117.44	112.17	121.90		123.52
010	103.20	105.25	108.14	109.30	
011	114.48	81.98	108.11	104.38	
012	93.79	92.70	103.09	90.73	
013	108.27	102.15	109.59	110.38	
014	133.72	128.88	128.63	113.06	
015	98.66	84.09	99.94	88.36	
017	115.39	96.69	114.82	94.95	
018	101.44	70.19	120.57	103.15	
019	120.54	108.55	115.19	116.33	
020	86.75	78.94	93.82	89.73	
021	111.52	95.01	110.52	70.73	
022	101.63	114.59	118.98	109.46	
023	110.94	100.51	108.89	108.24	
025	121.17	124.26	120.71	110.78	
