# Supplementary material for: AltitudeOmics: The Integrative Physiology of Human Acclimatization to Hypobaric Hypoxia and Its Retention upon Reascent
Source: PLoS One. 2014 Mar 21;9(3):e92191. doi: 10.1371/journal.pone.0092191 (PMC3962396; doi:10.1371/journal.pone.0092191)
Supplement: Table S5 — Peak Power Output and Submaximal Exercise Performance. Individual maximal exercise performance and 5-km time to completion data at SL, ALT1, and ALT16 and field exercise testing results at SL, ALT1, ALT16, POST7 and POST21. (PDF) [file pone.0092191.s005.pdf]

Table S5. Peak Power Output and Submaximal Exercise Performance

ID	VO_{2max} (l/min)			Peak Power Output (W)			5km Time to Completion (s)			Running speed (m/s)				
	SL	ALT1	ALT16	SL	ALT1	ALT16	SL	ALT1	ALT16	SL	ALT1	ALT16	POST7	POST21
001	3.52	2.60	2.95	310	190	227	480	800	601	3.19	1.46	1.78		1.74
002	4.07		2.95	353		250	484		587	3.79	2.18	2.16		2.21
003	2.04		1.59	160		100	677		881	2.21	1.43	1.40		
004	4.28		2.80	327		190	484		613	3.83	2.00	2.14		2.03
005	2.24	1.56	1.87	181	130	130	636	853	789	2.35	1.45	1.45		1.39
006	3.57		2.70	265		160	566		746	2.92	1.79	1.98		1.82
007	5.00		3.23	330		250	489		655	3.55	1.94	2.22		2.14
010	2.59	2.00	1.88	225	160	160	700	805	791	2.89	1.43	1.44	1.44	
011	3.46	2.13	2.47	239	160	160	558	653	659	2.78	1.66	1.70	1.76	
012	3.52	2.49	2.52	268	160	160	557	641	719	2.51	1.53	1.49	1.76	
013	3.64	2.92	3.07	285	212	212	558	642	706	2.68	1.67	1.84	1.80	
014	4.75	3.26	2.72	330	235	220	531	646	601	2.80	1.54	1.79	1.80	
015	3.14	1.35	1.46	213	100	100	586	856	807	2.86	1.49	1.81	1.63	
017	3.19	2.01	2.13	232	145	145	568	706	988	2.29	1.20	1.36	1.37	
018	3.40	2.59	2.99	257	167	160	539	627	684	2.70	1.55	1.72	1.54	
019	2.72	1.74	1.87	220	130	145	581	733	800	1.91		1.16	1.13	
020	2.18	1.93	2.03	220	160	160	715	763	748	2.77	1.47	1.46	1.34	
021	4.27		2.99	300	205	198	497	637	573	3.16			1.91	
022	4.10	3.05	2.66	302	212	220	531	650	655	3.31	1.65	2.00	1.95	
023	3.75	3.06	2.93	355	235	235		599	618	2.86	1.78	1.90	1.79	
025	2.38	1.89	2.16	198	130	130	643	733	789	2.29	1.36	1.42	1.55	
